# Spatiotemporally programmed dielectric liquid crystal elastomer: Electro-reversible 3D morphing via inverse 4D printing

**DOI:** 10.1126/sciadv.aeb2289

**Published:** 2025-11-26

**Authors:** Huiyao Zhao, Zike Chen, Jiahao Li, Yingwu Luo, Zhike Peng, Guoyong Mao, Rui Xiao, Jie Mao

**Affiliations:** ^1^ School of Chemistry and Chemical Engineering, Ningxia University, Yinchuan 750021, China.; ^2^ State Key Laboratory of Fluid Power and Mechatronic Systems, Department of Engineering Mechanics, Zhejiang University, Hangzhou 310027, China.; ^3^ State Key Laboratory of Chemical Engineering, College of Chemical and Biological Engineering, Zhejiang University, Hangzhou 310058, China.; ^4^ School of Mechanical Engineering, Ningxia University, Yinchuan 750021, China.

## Abstract

Programmable shape-morphing materials offer transformative potential in soft robotics and biomedical engineering, yet achieving reversible and precise control over complex 3D deformations remains a notable challenge due to the difficulty in spatially programming nonlinear mechanics. Here, we introduce a shear-assisted digital light-processing 4D printing strategy for spatiotemporal programming the mechanical anisotropy of dielectric liquid crystal elastomers (DLCEs). The printed DLCE actuators exhibit reversible multidimensional shape morphing (e.g., bending, twisting, and complex curved surfaces) under electric fields, governed by regional stiffness gradients. An inverse design algorithm is also developed to convert target 3D surfaces into executable printing tasks. Submillimeter-scale fidelity in reconstructing complex geometries, such as a panda face, a biomimetic plant and the Yellow River’s landform, demonstrates capabilities applicable to soft robotics and adaptive systems.

## Teaser

4D printing of dielectric liquid crystal elastomers enables spatiotemporally programmed shape morphing.

## INTRODUCTION

Stimuli-responsive materials capable of programmable 2D-to-3D shape transformations have enabled transformative advances in flexible electronics, soft robotics, and biomedical engineering (*1*–*5*). These multidimensional, shape-shifting systems offer innovative design paradigms for adaptive structures across scales, from macroscale deployable devices (e.g., reconfigurable aerial systems) to microscale functional components, such as micro/nano-actuators and sensors (*6*–*14*). Their ability to optimize locomotion, enhance environmental adaptability, and integrate multifunctionality underscores their technological significance (*10*, *15*–*22*). Despite progress in soft-matter transformations (*1*, *23*), achieving reversible and precise control over complex shape-morphing systems remains a significant challenge, stemming from the difficulty in spatially precise programming of nonlinear mechanics and actuation topology construction, as well as in managing the numerous sophisticated deformation configurations that soft materials may exhibit under external stimuli (*24*–*27*).

Recent strategies use smart materials and thin-film architectures with spatiotemporally modulated geometries, tunable stiffness, and programmed strain fields to achieve controllable morphing (*1*, *11*, *28*–*32*). For instance, liquid crystal elastomers (LCEs) (*33*) excel in achieving preprogrammed, reversible morphing through molecular alignment and are typically driven by thermal or photonic stimuli, providing high work density and complex shape changes. However, their response speed is generally slow, and precise spatiotemporal control in real-time remains challenging. Magneto-active elastomers (*34*) enable untethered, fast, and reversible transformations with excellent penetration in various environments, yet they often require incorporating rigid magnetic particles that may compromise the material’s compliance and homogeneity. In addition, achieving highly localized control of magneto-active elastomers can be complex due to the low resolution of the driven magnetic field. Other systems, such as hydrogels, shape memory polymers, and electrochemical actuators, also offer unique capabilities but face their own constraints in terms of response speed, environmental requirements, or integration complexity.

Dielectric elastomers (DEs), one of the electroactive soft materials that deform their shape under an electric field via Maxwell stress, stand out for their rapid response, remote controllability, and highly programmable actuation modes (*9*, *35*–*42*). Typical 3D morphing in DE actuators (DEAs) relies on spatially patterned electrodes and anisotropic mechanical constraints, often fabricated using template-guided methods (*37*, *38*, *40*, *42*). While this template-assisted localized actuation approach is promising, recent advances in additive manufacturing and micro assembly techniques have expanded design possibilities (*30*, *35*, *38*, *40*, *42*). For example, multicore-shell direct ink writing (DIW) 3D printing technology has been used to fabricate DEAs with sophisticated architectures, while DIW technology still suffers from limitations such as a relatively low printing resolution (around 400 μm) and the presence of interfacial defects (*8*, *9*). Moreover, achieving complex 3D curved surfaces with DEAs remains a considerable challenge. Key obstacles include the lack of efficient methods to spatiotemporally program mechanical topologies governing electro-actuation stress distribution, as well as the extensive computational cost and limitations of intuition-based design for intricate deformations (*37*, *42*).

**Fig. 1.**
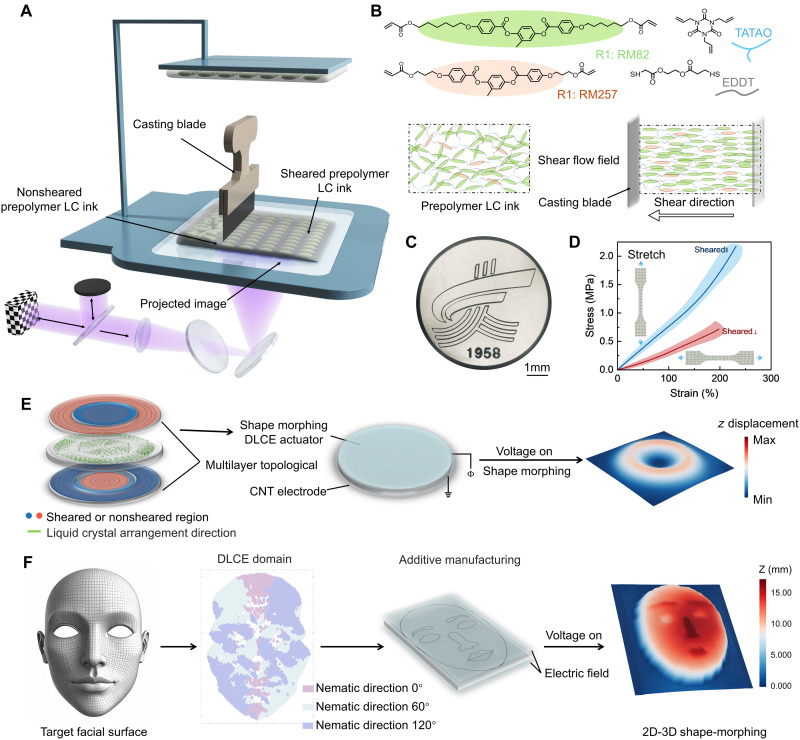
Template-free programming and inverse design of spatially distributed mechanical anisotropy in DEs, enabling reversible 3D shape morphing under electric actuation. (**A**) Schematic of shear-induced alignment of liquid crystal units in 4D printing. (**B**) Chemical structures of liquid crystal monomers (RM257 and RM82), flexible linker (EDDT), and cross-linking agent (TATATO) used in synthesizing dielectric LCEs (DLCEs). (**C**) Optical microscopy image of Ningxia University emblem–shaped DLCE samples between 0° and 90° polarizers (scale bars, 1 mm). (**D**) Stress-strain curves of DLCEs that are shear-printed parallel and perpendicular to the direction of the axial stress in tensile testing. (**E**) Schematic representation of the programming approach for achieving complex shape morphing in DLCEs. (**F**) Schematic representation of our inverse design approach for achieving complex three-dimensional facial deformations in DLCE.

To address this gap, we introduce a template-free programming and inverse design strategy enabling spatially programmed mechanical properties in DEs for dynamic, reversible 3D morphing under electric actuation. Using a photocurable elastomer with stiff liquid crystal mesogens, we developed a shear-assisted digital light processing (DLP) 4D printing method incorporating a movable scraper to fabricate dielectric LCEs (DLCEs) (Fig. 1, A and B). This approach actively tunes local network topology via printing parameters. Embedded shear flow fields precisely orient mesogens (Fig. 1, C and D), enabling ultraviolet (UV)–cured multilayer structures with spatiotemporally programmable mechanical heterogeneity. Compared to the DIW-based 3D printing approach for fabricating LCEs (*7*), our strategy demonstrates significant advantages in terms of printing resolution, order degree of mesogen, interfacial bonding quality, and manufacturing speed. Since regional stiffness gradients govern localized electromechanical deformation, DLCE actuators with such architectures achieve reversible complex 3D transformations through encoded mechanical properties under electric fields (Fig. 1E). We further established an inverse design strategy that converts target 3D surfaces into executable printing tasks. This framework determines optimal mechanical property distributions in DLCE films for fabrication via our shear-assisted DLP 4D printing. By converting the high-dimensional challenge of 3D anisotropy programming into a tractable 2D parameterization task, this approach significantly reduces computational complexity while enabling high-fidelity shape replication under actuation. Various complex 3D shapes and Gaussian curved surfaces, including facial models (Fig. 1F), a biomimetic plant [Graptoveria Amethorum (GA)], and a topographic map of the Yellow River, can be successfully constructed at a millimeter scale using our technology, showing promising application potential in bioinspired soft robotics, microfluidic systems, and biomedical engineering.

## RESULTS

### Localized programming of liquid crystal mesogens via shear-assisted printing

The one-pot thiol-acrylate/thiol-ene click reaction strategy was used to formulate the DLCE liquid resin system (*43*, *44*), selected for its rapid kinetic characteristics. The formulated ink consists of UV-responsive thiol-terminated oligomers specifically designed to achieve controlled cross-linking during photopolymerization, ensuring precise molecular alignment and effective curing during printing (note S1). The π-π conjugated structure in liquid crystal molecules endows them with rigid rod-like characteristics, enabling orientation along their long axis under shear forces (*45*). During the shear-assistant 4D printing process, the integrated blade applies necessary shear forces for liquid crystal alignment, where controlled shear flow fields enable precise molecular orientation that is subsequently fixed via UV curing. The combination of controlled shear flow fields and patterned UV curing mechanisms allows for high spatiotemporal precision in topological structure programming. The submillimeter-scale printed sample of the Ningxia University emblem shown in Fig. 1C fully demonstrates the exceptional precision and versatility of this printing technology for fabricating complex geometric structures.

**Fig. 2.**
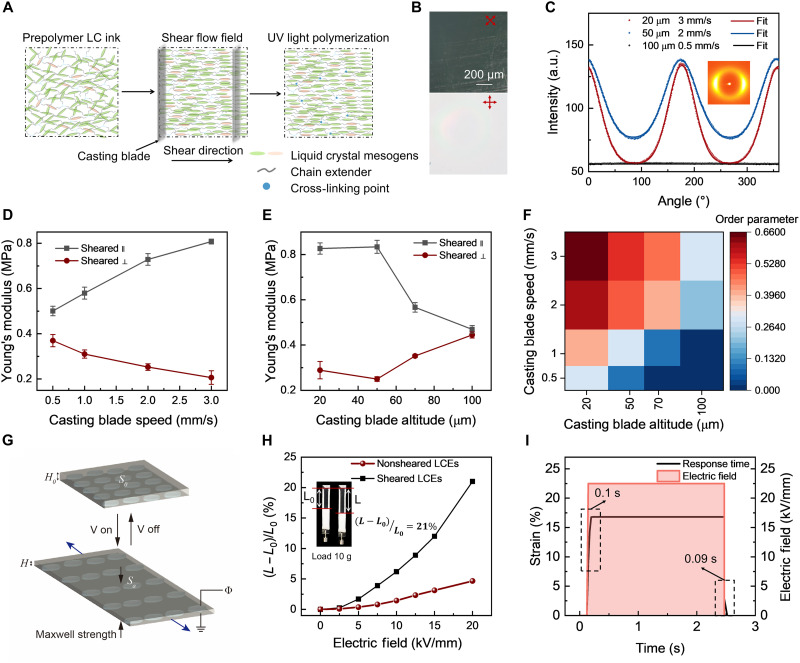
Structural and electromechanical characterization of the DLCE film. (**A**) Schematic diagram of molecular mesogens of DLCEs during blade coating and after solidification. (**B**) POM images of a printed DLCE film showing birefringence caused by shear-induced alignment. (**C**) 2D wide-angle x-ray scattering patterns of DLCE samples manufactured under different printing parameters (blade altitude of 100 μm and blade speed of 0.5 mm/s, blade altitude of 20 μm and blade of speed 3 mm/s). Normalized intensity is plotted as a function of the azimuthal angle of DLCE samples fabricated under different printing parameters. (**D**) Young’s modulus of DLCEs that are parallel shear printed and perpendicular shear printed the direction of the axial stress in tensile testing at different casting blade speeds. (**E**) Young’s modulus of DLCEs that are shear-printed parallel and perpendicular to the axial stress direction in tensile testing at different casting blade altitudes. (**F**) Order parameter of DLCE samples fabricated under different printing parameters. (**G**) Schematic illustration of electro-actuation in DLCEs. (**H**) The actuation strain versus electric field strength curve for the dielectric DLCE actuator was measured on a specimen prestretched under a load of 10 g. (**I**) Response and recovery time of the dielectric DLCE linear actuator.

During printing, the viscosity of the prepolymer, molecular weight, and cross-linker content are critical parameters that determine whether the shear flow field can provide sufficient moment of inertia for molecular alignment and effectively lock the liquid crystal arrangement within the relaxation time (fig. S6). On the basis of this, we optimized the physicochemical properties of the prepolymer and ultimately selected a formulation containing 50 wt % solvent, a thiol:acrylate ratio of 20:18, and 0.13 wt % cross-linker (detailed in note S1). To precisely control the direct shear force applied to the prepolymer, the doctor blade speed and height were determined to achieve optimal local alignment accuracy for the liquid crystals (Fig. 2A). Using these optimized parameters, we further evaluated printing precision by fabricating various micrometer-scale stripes, all of which exhibited pronounced birefringence under polarized microscopy (Fig. 2B), confirming excellent alignment order at micrometer-level precision (~150 μm) (fig. S8A). Under controlled curing depth conditions (a maximum blade height of 100 μm), increasing the shear rate significantly enhances molecular orientation, while a larger blade height reduces anisotropy due to shear stress dispersion, endowing the material with distinctive load-responsive characteristics. Two-dimensional wide-angle x-ray diffraction (WAXD) and polarized optical microscopy served as key characterization methods. The WAXD data revealed that as the blade speed increased and height decreased, the DLCE samples exhibited a transition from isotropic rings (preprogramming) to two distinct arcs perpendicular to the programming stress direction (postprogramming) at ambient temperature (Fig. 2C). By processing the WAXD data into normalized intensity-azimuthal angle distribution curves and calculating the order parameter (Fig. 2, D and E), it is found that at a blade height of 20 μm and a moving speed of 3 mm/s, the liquid crystal order parameter improved from <0.1 (isotropic) to 0.66 (Fig. 2F). Considering both fabrication efficiency and curing depth, the optimal printing parameters were chosen as: a blade height of 50 μm and a moving speed of 3 mm/s.

A typical DLCE with well-defined nematic phases exhibits pronounced mechanical anisotropy. Stress-strain measurements revealed a Young’s modulus of 230 ± 10 kPa perpendicular to the director axis, which is comparable to conventional DEs. In contrast, the parallel direction showed a significantly higher modulus of 809 ± 12.5 kPa. This disparity originates from the preferential alignment of polymer chains along the director axis, and the anisotropic mechanical properties can be further tuned by the shear printing parameters (fig. S9). Figure S8B presents optical micrographs of the printed DLCE under crossed polarizers (0° and 90°) before and after stretching, demonstrating the sample’s excellent elasticity and high-contrast birefringence patterns, along with dynamic birefringence phenomena under tensile stress.

The printed DLCE thin films exhibit a high dielectric constant (6.8) perpendicular to the liquid crystal domain alignment direction, which is much higher than that of most conventional DEs, such as 4.7 for 3M VHB tapes (fig. S9C). Coupled with their unique anisotropic mechanical properties, the DLCE film can be highly deformed anisotropically under the electric field due to the Maxwell stress (Fig. 2G). As shown in Fig. 2H, the DLCE can achieve up to 21% uniaxial strain response, which is four times larger than that of isotropic DLCE films. These exceptional actuation properties establish a material foundation for programming complex deformations (Fig. 2I).

### Printing developable surface deformation under electric field

**Fig. 3.**
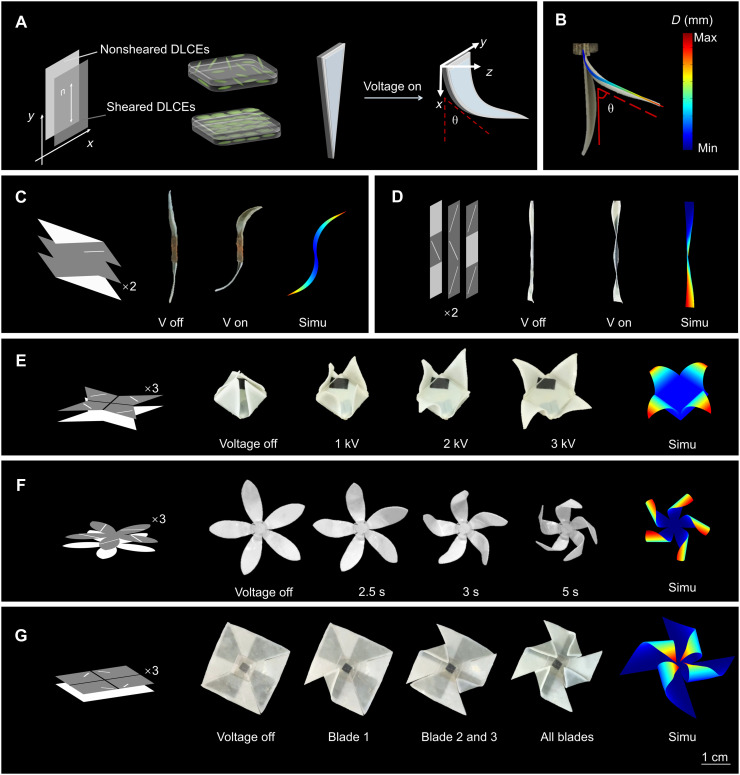
Bending units with precise controls of the developable surfaces in actuation. (**A**) Principle of bending in the printed DLCE actuators. Light gray and dark gray represent nonsheared and sheared DLCE layers, respectively, and the multilayer mechanism consists of a bending unit, actuating principal schematic (n respects nematic direction of sheared DLCEs) (**B**) The bending actuator experimental and simulated images. (**C** to **G**) Complex shape-morphing actuators with experimental images (the superscript ×2 indicates a bilayer stacked configuration) and FEM. (C) The “S” shape actuator. (D) The twisting actuator. (E) The pyramid actuator. (F) The flower actuator. (G) A windmill actuator. (scale bars, 1 cm).

Developable surface deformation, such as twisting, origami-like folding (*46*), and bio-inspired shape changes can be decomposed into numerous simple bending actuation units (*1*, *2*, *16*, *29*, *30*, *36*). To validate the efficacy of our shear printing strategy, we first use synthetic DLCEs to design a fundamental bending actuation unit composed of two-layer topological structures with nonsheared and sheared DLCE layers (figs. S10 and S11), represented by light gray and dark gray, respectively, as shown in Fig. 3A. The double-headed vector **n** denotes the nematic director of sheared DLCEs. Upon applying an electric field, both layers contract along the thickness *z* direction, while the sheared DLCE layers exhibit larger unidimensional strain due to their intrinsic electromechanical anisotropy compared to the nonsheared layers, resulting in a strain mismatch between the two layers, and further leading to an out-of-plane bending actuation, as shown in Fig. 3A and fig. S12. The maximum bending angle of 70° can be achieved via tuning the thickness of sheared and nonsheared layers (fig. S13 and movie S1). Notably, the response time is within 0.17 s and a full recovery occurs in approximately 0.07 s, which is much faster than the other conventional stimuli-responsive materials, like thermal-responsive or light-stimulated and pneumatic-driven actuators (*47*) (fig. S14), highlighting the potential for high-speed applications of DLCEs. In addition, this unit could bend over 20,000 working cycles (fig. S15), confirming its consistent and repeatable long-life actuation performance.

To predict the actuation behavior of DLCE with programmed nematic orientation domains under an electric field, we developed an electromechanical coupled model (*48*) with the detailed modeling and simulation provided in note S2. This model incorporates the anisotropic mechanism (*47*) arising from the constraints imposed by nematic liquid crystal mesogens, enabling simultaneous capture of mechanical and electroactive responses of both parallel and perpendicular to the nematic orientation (figs. S20 and S21) (*49*). The model was implemented into COMSOL Multiphysics, a commercial finite element suite. For nonsheared DLCEs, considering its isotropic mechanical response and small actuated strain remaining within the linear elastic regime, a linear elastic model is adopted, while the dielectric response is kept with a recalibrated dielectric permittivity from experimental data (detailed in note S2). As shown in Fig. 3B, the simulation results show good agreement with the electro-actuation experiments, which confirms the accuracy of the model and validates the applicability of the numerical simulations for guiding the design of complex shape morphing via programming liquid crystal mesogen orientations.

To show the capability of designing developable surfaces, we demonstrated more complex developable deformation architectures through the bending unit assembly and simulation protocols (Fig. 3, C to G). First, an S-shaped actuation mode was constructed using bidirectional bending units, where asymmetric configuration of nonshearing layers controlled bending direction, enabling reversible transformation from planar to doubly curved S-shaped structures under an electric field of 20 kV/mm (Fig. 3C). We further developed a trisecting rectangular actuator with liquid crystal molecules aligned perpendicular to diagonals in each region, combined with alternately arranged nonshearing layers, which exhibits unique deformation from local curling to global torsional angular displacement under electric stimuli (Fig. 3D and figs. S20 and S21). A four-unit triangular bending actuator can successfully present the opening process of a pyramid structure, while the developable surface can be controlled by the applied voltage (Fig. 3E). To mimic the blooming behavior of flowers, a blooming actuator with four elliptical units of 45° director alignment of liquid crystal mesogens has been designed, which precisely replicated the Trachelospermum leaf morphology (Fig. 3F). Moreover, we demonstrated an origami-like folding actuation of windmill configurations, where every blade of windmill can be activated by electric field solely and controlled remotely, showing the remote programming capability of DLCE shape morphing actuators via electric signals (Fig. 3G). Furthermore, the close agreement between simulations and experimental observations of the above actuators validates the reliability of our finite element method, which confirms that our anisotropic electromechanical model can effectively guide the design of complex deployable structures through precise spatiotemporal programming of DLCE geometry and local director fields (fig. S24 and movie S2).

### Programming nondevelopable surface deformation

**Fig. 4.**
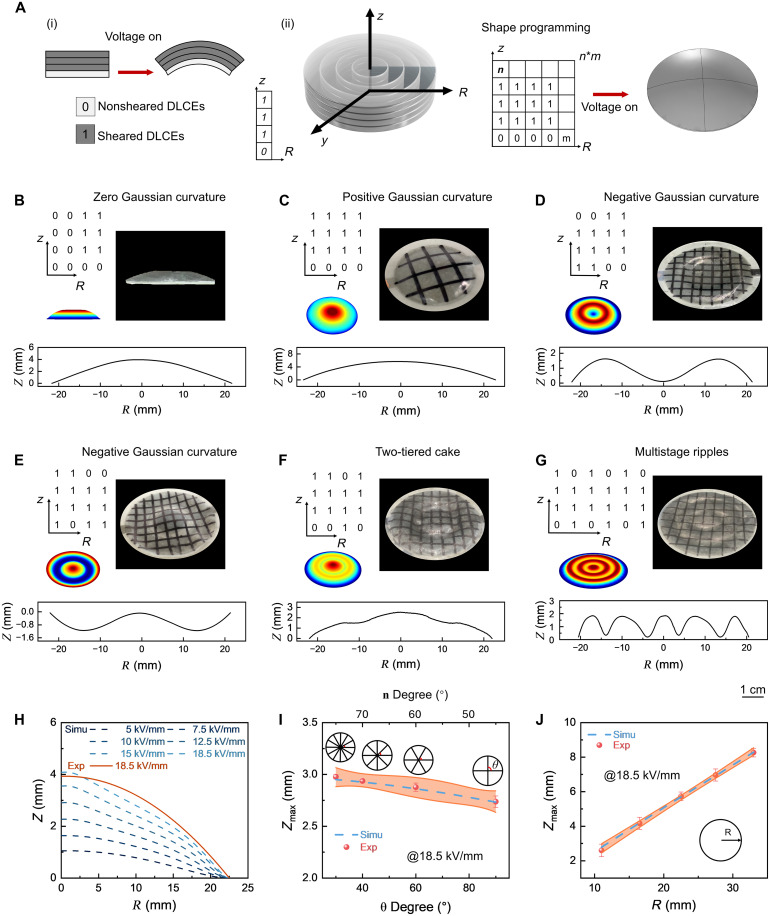
Programming Gaussian curvature and other nondevelopable surfaces by 4D printing. (**A**) Schematic of a circular actuator programmed via a matrix, where 0 and 1 denote nonsheared and sheared DLCEs, respectively. (**B**) Programming matrix for zero Gaussian curvature (top left), simulated (bottom left), experimental images (right), and actuation profiles (bottom). (**C**) Positive Gaussian curvature. (**D**) Negative Gaussian curvature. (**E**) Alternative negative Gaussian curvature. (**F**) Two-tiered cake actuation. (**G**) Multistage ripples actuation. (**H**) Simulated actuating profiles of positive Gaussian curvature actuators upon various electric fields and experimental actuating profiles at 18.5 kV/mm. (**I**) Simulated maximum displacement ($z_{\text{max}}$) of positive Gaussian curvature actuators at different centroid angles (θ) and experimental actuating $z_{\text{max}}$ at 18.5 kV/mm. (**J**) Simulated $z_{\text{max}}$ of positive Gaussian curvature actuators at varying radii (*r*) and experimental actuating $z_{\text{max}}$ at 18.5 kV/mm.

In addition to designing developable surfaces, our strategy of printing electrically active surfaces can be further extended to complex nondevelopable shape morphing structures, through the precise spatiotemporal programming of liquid crystal mesogen director alignment in DLCE actuators (fig. S26). Inspired by Alan Turing pattern of morphogenesis and pattern formation, we designed a binary classification framework for DLCE shape morphing actuators (“0” for nonsheared region and “1” for sheared region) to mimic the discrete-state systems, where local interactions (sheared/nonsheared states) give rise to activated global geometric complexity (Gaussian curvature distributions), thereby enabling programmable deformation with spatial differentiation. To validate the efficacy of our printing strategy for building nondevelopable surfaces, we designed an actuator with a multilayer planar disk structure that transforms into fundamental nondevelopable shapes like zero, positive, and negative Gaussian curvature surfaces (fig. S27). In these actuator configurations, each annulus is indexed as a row (*m*) along the radial direction (*r*), and each layer along the *z* axis is indexed as a column (*n*), forming an *m* × *n* matrix (Fig. 4A). Every matrix element features independent spatiotemporal programmability, allowing the actuator to accurately realize surfaces with varying Gaussian curvatures. For the Gaussian curvature, *Z* (out-of-plane displacement) and *R* (radius) describe the initially flat elastomer disk, and *R*_max_ is fixed at 22.5 mm for manufacturing considerations.

As for a truncated conical actuator with zero Gaussian curvature (Fig. 4B and movie S3), the local out-of-plane actuation unit is configured as (1, 1, 1, 0)*^T^*, whereas the region with zero out-of-plane displacement is set as (0, 0, 0, 0)*^T^* (Fig. 4B, top left). When actuated under the electric field, this actuator deforms from a flat disk into a circular truncated cone, where the magnitude of the gradient vector $\left[\nabla f=\left(\partial z/\partial x,\partial z/\partial y\right)\right]$ of the profile remains constant. To achieve out-of-plane deformation of positive Gaussian curvature configurations (Fig. 4C and movie S3), where the gradient vector magnitude exhibits radial enhancement, a DLCE actuator incorporating four (1, 1, 1, 0)*^T^* unit cells was designed (Fig. 4C, top left). For the negative Gaussian curvature shapes of the DLCE actuator (Fig. 4D and movie S3), the magnitude of the gradient vector is the highest near the center and edges, while it diminishes at a distance of *R*/2 from the center. This radial actuation is facilitated by two sets of DLCEs with complementary orientations: (1, 1, 1, 0)*^T^* and (0, 1, 1, 1)*^T^*, where the displacement field at the center (*z* increasing with radius) opposite to that at the edges (*z* decreasing with radius) (Fig. 4D, top left). The out-of-plane deformation direction of negative Gaussian curvature shapes can be varied by tuning the arrangement of nonsheared/sheared regions (Fig. 4E and movie S3). Furthermore, more intricate surfaces like two-tiered cake (Fig. 4F, fig. S28, and movie S3) and multistage ripples (Fig. 4G and movie S3) can be created by designing the *m* × *n* matrix and precisely programming the nonsheared/sheared regions, confirming the viability of developing complex surfaces via our printing approach. All the aforementioned actuator profiles closely match the simulated results (Fig. 4C, lower left and right).

The displacement of printed DLCE shape morphing actuators is mainly determined by three key control parameters: applied electric field, actuator radius, and localized liquid crystal mesogen orientations. To validate the simulation results of the model, the actuator deformed into positive Gaussian curvature configurations is chosen to be further analyzed. The simulated and experimental actuation profile curves agree well across a range of electric fields. A higher electric field results in increased maximum *z* axis displacement $z_{\text{max}}\;$ (Fig. 4H and fig. S29), demonstrating the quantitative deformation controllability of DLCE actuators via electric signals. The nematic direction of liquid crystal molecules within the DLCE actuator is also crucial for determining the extent of actuation. We calculated the relationship between the angle of the liquid crystal nematic director **n** and $z_{\text{max}}$. The angle between the nematic director **n** and the positive direction of the *x* axis is $\left(\text{180}{}^{\circ}+\theta \right)/2$, which can be substituted by the centroid angle θ of the circular actuator equivalently. Both simulations and experiments indicate that $z_{\text{max}}$ increases as the angle of the liquid crystal nematic director decreases (Fig. 4I and fig. S30). In addition, the actuator’s size also significantly influences the actuation magnitude. Our designs of actuators revealed that $z_{\text{max}}$ increases with radii, showing positive feedback in both experimental and simulated results (Fig. 4J). This allows us to tailor the radius of actuator to achieve a specific target displacement.

### Inverse design for complex surface deformation

In practical applications, target deformation structures often exhibit complex nonaxisymmetric features. This necessitates the development of a robust inverse design strategy with the capability of simultaneously programming both the spatiotemporal nematic director field and the initial planar geometry with high precision, a significant challenge to accomplish solely through intuitive design. To address this, we developed an inverse design strategy to program liquid crystal mesogens orientations of DLCEs, enabling electrically actuated transformation into desired 3D curved shapes.

**Fig. 5.**
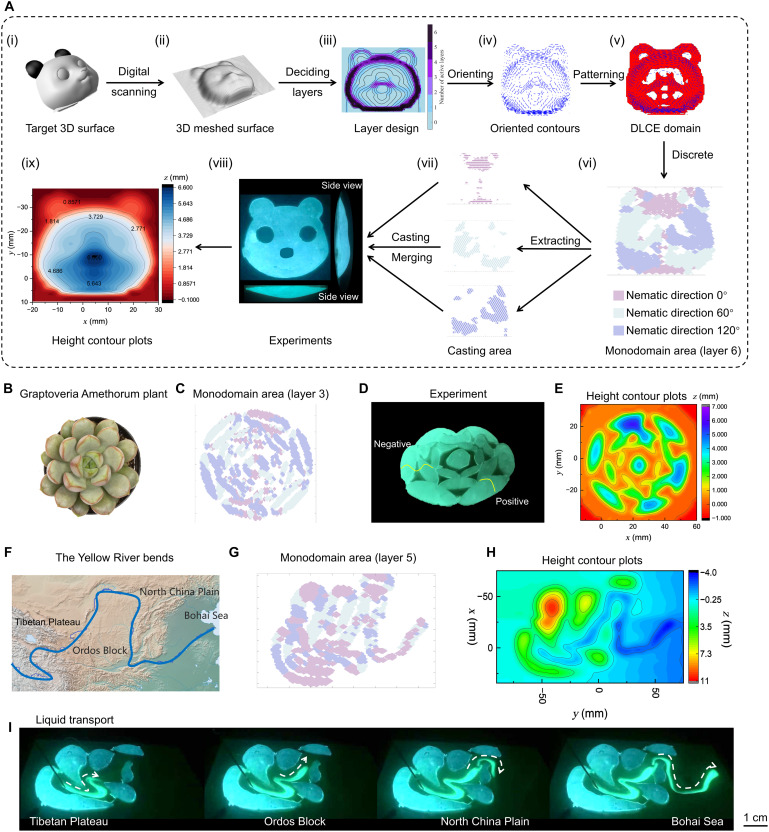
Inverse design of complex nondevelopable surfaces. (**A**) (i) A 3D target panda face shape. (ii) Discretize the target shape to obtain the target coordinate information set (*x*, *y*, and *z*). (iii) Spatial distribution of the required number, Nm, of sheared DLCE layers. (iv) Oriented contour line segments with angles between the positive *x* axis: 0°, 60°, and 120° determined by the chosen bending unit. (v) Overlay each layer of sheared DLCE pattern with oriented contour lines to split the pattern for manufacture. (vi and vii) Divide the sheared DLCE pattern into three categories based on the angles of segments. Each category decides the casting area for the corresponding nematic direction. (viii) The activated shape in experiment. (ix) The activated height contour plot. (**B**) Target Graptoveria Amethorum (GA) shape in nature. (**C**) Inverse-designed casting areas for the GA shape. (**D**) Activated shapes of imitating the GA-DLCE structure in experiments. (**E**) The activated DLCE structure height contour plot. (**F**) Target shape of the landforms of the Yellow River bend. (**G**) Inverse-designed casting areas for the Yellow River bend. (**H**) The activated DLCE structure height contour plot for the Yellow River bend. (**I**) Activated shapes for the Yellow River, achieving a liquid transport function. The natural streamline shape of the Yellow River is reproduced as a result of the inverse design.

As shown in Fig. 5A (i) and (ii), we take a 3D panda face as the target surface as an example. After obtaining the coordinate data z(X,Y) of the target surface from a laser scan of the target surface, we compute the contour lines [black lines in Fig. 5A (iii)] using the built-in function contour in MATLAB. We then calculate the distribution for the number of monodomain DLCE layers via the mapping transports method (detailed in note S3) and the spatiotemporal nematic director field of liquid crystal mesogen, which is discretized from continuous contour lines. The distribution for the number of monodomain DLCE layers controls the local electric potential and, consequently, the required stretch ratio for the target shape. Meanwhile, the nematic director field decides the local deformation directions. Under the combined action of the layer distribution and the director field, the planar DLCE configuration can achieve versatile local deformations, ultimately morphing into our desired shape. To simplify the printing process, all oriented contour lines are categorized into three directional classes: 0°, 60°, and 120° with respect to the *x* axis [Fig. 5A (iv)]. By integrating the oriented director field with monodomain layer design, we obtain the monodomain patterns that define the area and the nematic directions. Further, by using the Bresenham algorithm to identify the pixel grid that coincides with the oriented directors, which are represented by blue segments in Fig. 5A (v), the patterns can then be discretized into three related DLCE regions with programmed nematic orientations (distinguished by three colors) [Fig. 5A (vi)]. Extracting these three pattern regions with different colors, the slice pattern of each layer for the practical printing process can be created. The above procedure is programmed in MATLAB following a flowchart in fig. S34. As shown in Fig. 5A (viii) and (ix), the target panda surface is well reproduced, validating the applicability of our inverse design method for guiding the printing of a complex deformable surface in experiments. A more detailed description of our inverse design strategy can be found in the note S3.

To further demonstrate the capabilities of DLCE materials transforming into 3D shapes with intricate geometric features, we printed DLCE materials to replicate the morphology of a GA plant featuring 19 petals (Fig. 5B and fig. S35). Followed by our inverse design program, the localized shearing pattern (in layer 3) of monodomain DLCE can be identified (Fig. 5C), which was converted into the projection patterns of the printing process, and ultimately achieved an electrically controllable undulating surface with both positive and negative Gaussian curvatures (Fig. 5, D and E). Note that the discrete blue points within the green domains in Fig. 5C represent shearing areas caused by nonsmoothness in the scanned data. These points can be manually replaced with the surrounding colors in practice. Moreover, we also replicated the landforms of the Yellow River in China via our DLCE materials and inverse design program, which represents some of the most unpredictable and complex nondevelopable shapes in nature. By extracting the main protrusion features of the contour lines of the Yellow River, the DLCE orientation pattern (in layer 5) can be obtained, followed by our inverse design procedure (Fig. 5G). We then fabricated DLCE materials to electrically simulate the landform of the Yellow River. The height contour profiles of DLCE actuators present the undulation of the terrain, highlighting the difficulties in realizing complex shapes via intuitive design (figs. S36 to S38). Furthermore, the actuated landform-shape can realize liquid transport functionality along the height difference of terrain, mimicking the natural behavior of the Yellow River, demonstrating promising application potentials of our materials in bioinspired systems (movie S4). All of the complex nondevelopable surface structures have six layers in total, varying the number of sheared layers spatially calculated from the inverse design method. The applied electric field is set to 20 kV/mm for all demonstrations. The activated areas are painted with a highlighter pen to feature the actuation in experiments. Experimental actuation progress is included in the movie S4.

## DISCUSSION

This study demonstrates a template-free programming strategy combined with inverse design to achieve reversible 3D morphing in DLCEs. By leveraging shear-assisted DLP 4D printing, we enable precise spatiotemporal control over mechanical anisotropy through the localized alignment of liquid crystal mesogens. This approach eliminates traditional fabrication constraints and allows programmable stiffness gradients essential for complex actuation. The printed DLCE actuators exhibit exceptional multidimensional deformability, including both developable surfaces (e.g., bending, twisting) and nondevelopable Gaussian curvature geometries. Crucially, these transformations are electrically driven, reversible, fast (<0.2 s response/recovery), and fatigue-resistant (>20,000 cycles). Our inverse design algorithm successfully maps target 3D shapes to optimized 2D mechanical property distributions and executable printing tasks, enabling millimeter-scale fidelity in reconstructing intricate architectures like facial profiles (fig. S39), flora morphologies, and topographic maps. These capabilities highlight transformative potential in bioinspired applications, including soft robotics for adaptive locomotion, reconfigurable microfluidic systems, and minimally invasive biomedical devices. Future work will address scalability limitations and integrate multistimuli responsiveness to expand operational versatility. Despite these advances, the current system still faces limitations at the material level, including an inability to be reprogrammed, a lack of reconfigurability, and limited output force (fig. S40), while at the methodological level, it remains constrained by fixed boundary conditions and high computational costs. Future work will focus on overcoming scalability challenges and integrating multistimulus response mechanisms to further enhance its practical applicability.

## MATERIALS AND METHODS

Detailed materials and methods are provided in the Supplementary Materials.

### Experimental design

This study aims to validate the hypothesis that shear force–induced molecular orientation can effectively align liquid crystal mesogenic units in main-chain DLCE systems during layer-by-layer DLP printing without requiring pre-alignment treatments. To evaluate the efficacy of this fabrication approach, we designed, developed, and demonstrated a series of DLP-printed shape-morphing actuators capable of achieving complex deformations, including zero, positive, and negative Gaussian curvature configurations. Furthermore, leveraging inverse design strategies, we fabricated sophisticated topographical structures and successfully demonstrated their application in liquid transport.

### Preparation of DLCE liquid resin

To initiate the thiol-acrylate/thiol-ene click reaction, EDDT, RM257, RM82, and TATATO were dissolved in anhydrous tetrahydrofuran at controlled molar ratios within amber glass vials. The solution was then supplemented with 1 wt % TPO (a photoinitiator, 2,4,6-trimethylbenzoyldiphenyl phosphine oxide) and 2 wt % butylated hydroxytoluene to mitigate premature thermal polymerization. The reaction mixture was continuously stirred at 200 rpm on a heating plate maintained at 65°C for 4 hours to ensure complete oligomerization.

### DLP of DLCE actuators

All DLP-compatible design files were initially created using the CAD software SolidWorks. These files were subsequently imported into Autodesk Print Studio, where they were sliced into photo-pattern layers and converted into the “.ctb” format before being transmitted to the Autodesk Ember printer for fabrication. The oligomerized liquid resin was transferred to the resin vat and allowed to settle for 5 min to minimize air entrapment before initiating the printing process.

For thin structures [thickness (*h*) less than 50 μm, fewer than 10 layers], the printer’s in-plane (*xy*) resolution was measured at 150 μm, achieved using a 1280 × 800 pixel UV projector with a maximum build area of 70 mm by 40 mm. For thicker structures (50 μm < *h* < 200 μm, 10 to 40 layers), the resolution was adjusted to a range of 150 to 300 μm because of stronger light scattering to ensure optimal layer adhesion and structural integrity. The axial (*z* axis) resolution of the printer was maintained at 50 μm throughout the fabrication process. Following printing, all specimens underwent a two-stage postcuring treatment: (i) bilateral UV exposure (λ = 365 nm, intensity = 25 mW cm^−2^, 9 s per side) to ensure complete cross-linking, followed by (ii) thermal annealing at 70°C under vacuum for 24 hours to eliminate residual solvents and optimize material dielectric properties.

### Electro-actuation test

Carbon nanotube electrodes were applied to both sides of the DLCE film and connected to an external high-voltage power supply using copper wires. The material’s electro braking behavior was then examined.

### Numerical simulation

We simulated the electromechanical response of the DLCEs with the finite element software COMSOL Multiphysics The constitutive relationship differs from sheared and nonsheared DLCE. For nonsheared DLCE, we used the isotropic linear elastic model with a Young’s modulus of 0.533 MPa and the idea dielectric model with a relative dielectric permittivity of 2.98. For sheared DLCE, we implemented the proposed anisotropic electromechanical coupled model (detailed parameters of the model are shown in note S2) into COMSOL by defining the free energy in the solid mechanical interface. We used the weak form partial differential equations (PDEs) in COMSOL to compute the changing electric field strength with the increase of applied voltage. The voltage was set as a Dirichlet boundary to denote the electric potential on the surface of electrode. The mechanical boundary conditions were equivalented according to the specific conditions in experiments, a fixed boundary was set on the top of the horizontal face of triangular actuator in Fig. 3A (iv) as an example. In fig. S21A, we showed the anisotropic electromechanical response curve comparing with the theoretical response to validate our implement.

## Supplementary Materials

### The PDF file includes:

Notes S1 to S4 Figs. S1 to S40 Legends for movies S1 to S4 References
