# Supplementary material for: Spatiotemporally programmed dielectric liquid crystal elastomer: Electro-reversible 3D morphing via inverse 4D printing
Source: Sci Adv. 2025 Nov 26;11(48):eaeb2289. doi: 10.1126/sciadv.aeb2289 (PMC12652243; doi:10.1126/sciadv.aeb2289)
Supplement: Supplementary file 1 — Notes S1 to S4 Figs. S1 to S40 Legends for movies S1 to S4 References [file sciadv.aeb2289_sm.pdf]

Science Advances
AAAS

Supplementary Materials for

Spatiotemporally programmed dielectric liquid crystal elastomer: Electro-reversible 3D morphing via inverse 4D printing

Huiyao Zhao *et al.*

Corresponding author: Guoyong Mao, guoyongmao@zju.edu.cn; Rui Xiao, rxiao@zju.edu.cn; Jie Mao, maojie@nxu.edu.cn

Sci. Adv. **11**, eaeb2289 (2025)
DOI: 10.1126/sciadv.aeb2289

The PDF file includes:

Notes S1 to S4
Figs. S1 to S40
Legends for movies S1 to S4
References

Other Supplementary Material for this manuscript includes the following:

Movies S1 to S4

Supplementary Note 1 Material Design and Optimization of Dielectric Liquid Crystal Elastomers

In developing the dielectric liquid crystal elastomer (DLCE) liquid resin formulation, we employed a one-pot thiol-acrylate/thiol-ene click reaction approach due to its rapid kinetics. The initial formulation combined mesogenic diacrylates, specifically 1,4-bis-[4-(3-acryloyloxypropyloxy)benzoyloxy]-2-methylbenzene (RM257) and 1,4-bis-[4-(6-acryloyloxypropyloxy)benzoyloxy]-2-methylbenzene (RM82), with the flexible dithiol linker 2,2′-(ethylenedioxy) diethanethiol (EDDT) and the trivinyl cross-linker 1,3,5-triallyl-1,3,5-triazine-2,4,6(1H,3H,5H)-trione (TATATO). Kinetic studies reveal that the dithiol monomer functions dually as a chain extender and a chain transfer agent. Moreover, the presence of pendant, unreacted thiol groups, contributes to an increased dielectric constant of the material (*44*, *50*). RM82:RM257 (75/25) shows a clear nematic behavior at room temperature (*51*). The formulated ink consists of UV-reactive thiol-terminated oligomers specifically engineered for controlled cross-linking upon photopolymerization, facilitating precise molecular alignment and effective curing during the printing process. Incorporation of 1.3% (wt%) diphenyl(2,4,6-trimethylbenzoyl) phosphine oxide (TPO) as a photoinitiator demonstrated remarkable photocuring efficiency (Fig. S1), achieving approximately 90% gelation conversion within a 3-second exposure to ultraviolet irradiation (λ = 405 nm, intensity = 11.4 mW cm^{-2}).

As the crosslinker content increased, the modulus both parallel and perpendicular to the LC alignment direction increased. At 0.1 mol% of crosslinker, the lack of crosslinking points resulted in insufficient crosslinking density to maintain the orientation of LC columns after shear. Crosslinker contents below 0.1 mol% failed to rapidly achieve a gel content exceeding 90%. At 0.13 mol%, the crosslinking points effectively stabilized the LC column alignment while retaining the highest amount of unreacted thiol groups, leading to a material with low modulus and high dielectric constant (Fig. S2). Therefore, 0.13 mol% was selected as the optimal crosslinker content.

By adjusting the thiol-to-acrylate ratio, we regulated the chain extension reaction to control the length of LC molecular chains and investigated the influence of molecular weight on mesogen alignment. As the ratio approached 1:1, the molecular weight of the prepolymer gradually increased, leading to longer molecular chains (Fig. S3). The higher molecular weight and chain elongation complicated the alignment behavior during shear flow, as the prepolymer's molecular weight affected both relaxation time and moment of inertia. Longer LC prepolymer chains induced greater entanglement, increasing the moment of inertia of rod-like LC molecules and slowing relaxation. Consequently, the difference in modulus between the parallel and perpendicular alignment directions progressively decreased (Fig. S4A and S4B). Additionally, as the number of unreacted thiol groups declined, both dielectric constant and dielectric loss exhibited a decreasing trend (Fig. S4C and S4D).

Furthermore, when the thiol-to-acrylate ratio approached 1:1, the number of available crosslinking sites for subsequent photopolymerization decreased, reducing crosslinking density and compromising the stability of LC mesogen alignment after shear (Fig. S4A and S4B). Thus, an optimal thiol-to-acrylate ratio of 20:18 was identified, providing superior LC mesogen alignment, higher dielectric constant, and suitability for subsequent experimental applications.

The three primary liquid crystal phases are: Nematic (N) - characterized by uniform alignment of mesogenic molecules along the director (orientation order only); Smectic A (SmA) - featuring a layered structure with both orientation and positional order; and Smectic C (SmC) - structurally analogous to SmA. Generally, smectic liquid crystalline elastomers exhibit greater actuation strain, lower elongation at break, higher modulus, and larger enthalpy changes compared to their nematic counterparts, which can be attributed to the typically higher order parameters of smectic phases. The multi-layered printing structure and the introduction of shear flow fields via doctor blades increase the distance between adjacent rod-like liquid crystals, enabling a comparative study between SmC and nematic DLCE actuators (Fig. S5A). The SmC phase exhibits lamellar structural characteristics, with its wide-angle X-ray diffraction (WAXD) pattern showing crystalline-like diffraction peaks in the $2\theta=2°-3°$ range in addition to a diffuse diffraction peak at $2\theta=20°$. During printing parameter optimization, we observed diffraction peaks matching SmC phase characteristics under printing conditions with a doctor blade speed of 3 mm/s and altitude of 50 μm (Fig. S5B). This finding effectively demonstrates the material's higher order parameters and greater deformation capability (*52*).

We introduced organic solvents to modulate resin viscosity. Rheological characterization revealed an inverse relationship between solvent content and viscosity, with viscosity showing a monotonic decrease as solvent concentration increased. The formulated ink predominantly exhibits Newtonian fluid behavior at low to medium shear rates, while demonstrating pronounced shear-thinning characteristics under high shear rates typical of DLP printing processes (Fig. S6). All organic solvents completely evaporate during post-printing drying without affecting the material's mechanical and dielectric properties (Fig. S7).

Supplementary Note 2 Anisotropic electro-mechanical coupled model for DLCEs

To characterize the anisotropic electric activated response under an electric field, we develop an electro-mechanical coupled model for DLCEs. Upon application of electric fields, the attraction of opposite charges on either side of DLCEs induces the so-called Maxwell stress, which compresses the elastomer along the thickness direction and expands in the vertical plane. Meanwhile, the confinement effect of nematic liquid crystals induces an anisotropic mechanical behavior in the vertical plane, since the elastomer possesses a larger modulus along the nematic direction $\boldsymbol{n}$ than that in the direction perpendicular to $\boldsymbol{n}$.

The deformation can be described by a smooth one-to-one mapping $\mathbf{x} = \chi(\mathbf{X}, t)$, in which $\mathbf{x}$ and $\mathbf{X}$ denote the position of a point in the current and reference configurations, respectively. The deformation gradient tensor $\mathbf{F}$ can then be defined as

$$\mathbf{F} = \mathrm{Grad}\chi . \tag{S.1}$$

Here, the symbol Grad denotes the gradient with respect to the referential point $\mathbf{X}$. Further, the right and left Cauchy-Green deformation tensors $\mathbf{C}$ and $\mathbf{b}$ are given by $\mathbf{C} = \mathbf{F}^{\mathrm{T}}\mathbf{F}$ and $\mathbf{b} = \mathbf{F}\mathbf{F}^{\mathrm{T}}$, respectively.

The contributions for mechanical response of DLCEs under electrical stimulation can be decomposed into three parts: pure hyperelastic part Ψ^{eq}, dielectric part Ψ^{elec} and confinement effect part Ψ^{ce} of nematic liquid crystals (*48*). To reinforce incompressibility, the total energy density is represented as

$$\Psi = \Psi^{eq} + \Psi^{elec} + \Psi^{ce} - p(J-1), \tag{S.2}$$

where p is a Lagrange multiplier with physical meaning as the hydrostatic pressure, and $J = \det(\mathbf{F})$. Anisotropic behavior arises mainly from the confinement part. Thus, after decomposition, the pure hyperelastic part can be assumed as isotropic with the energy as a function of $\mathbf{C}$. Here we adopt a phenomenological hyperelastic energy using an exponential function:

$$\Psi^{eq} = \frac{C_1}{2} I_c \left[\exp\left(\frac{I_1 - 3}{I_c} \right) - 1 \right], \tag{S.3}$$

where C_1 is the shear moduli, I_c is a model parameter controlling the strain stiffening effect, and $I_1 = \mathrm{tr}(\mathbf{C})$ is the first invariant of $\mathbf{C}$.

As for the electric part, based on the fact that the dielectric permittivity of DLCEs changes slightly when actuated (Fig. S16), the idea dielectric model is adopted as:

$$\Psi^{elec} = \frac{1}{2\varepsilon} J^{-1} \hat{\mathbf{D}}\mathbf{C}\hat{\mathbf{D}}, \tag{S.4}$$

where $\varepsilon = \varepsilon_r \varepsilon_0$ denotes the dielectric permittivity constant and ε_r is relative permittivity, $\varepsilon_0 = 8.854187817 \times 10^{-12}$ F/m is vacuum permittivity constant, $\hat{\mathbf{D}}$ is referential electric displacement induced by an applied referential electric field $\hat{\mathbf{E}}$.

The confinement part induced by nematic liquid crystals is modelled as a fiber-like contribution to the free energy density. Allahyari and Asgari (*49*) developed a constitutive model for fiber-reinforced dielectric elastomers, where they take a series of invariants related to unit fiber directions to describe the contributions from fibers. The model was validated with experimental results. Here, we assume the free energy density is a function of the invariant $I_4 = \boldsymbol{n}\mathbf{C}\cdot\boldsymbol{n}$ with $\boldsymbol{n}$ denoting the unit nematic direction. The free energy density is then written as

$$\Psi^{ce} = \frac{k}{4}\left(I_4 - 1\right)^2 . \tag{S.5}$$

The thermodynamics require the following equations hold

$$\mathbf{S} = 2\frac{\partial \Psi}{\partial \mathbf{C}}, \tag{S.6}$$

$$\hat{\mathbf{E}} = \frac{\partial \Psi}{\partial \hat{\mathbf{D}}}, \tag{S.7}$$

where $\mathbf{S}$ is the second Piola-Kirchhoff stress, and $\hat{\mathbf{E}}$ is the nominal electric field. The Cauchy stress $\boldsymbol{\sigma}$ and the first Piola-Kirchhoff stress $\mathbf{P}$ has a relation with the second Piola-Kirchhoff stress as

$$\boldsymbol{\sigma} = \frac{1}{J}\mathbf{F}\mathbf{S}\mathbf{F}^{\mathrm{T}}, \tag{S.8}$$

$$\mathbf{P} = \mathbf{F}\mathbf{S}. \tag{S.9}$$

Using the chain rule of differentiation, it could be found that

$$\frac{\partial \Psi}{\partial \mathbf{C}} = \frac{\partial \Psi}{\partial I_1}\frac{\partial I_1}{\partial \mathbf{C}} + \frac{\partial \Psi}{\partial I_4}\frac{\partial I_4}{\partial \mathbf{C}}, \tag{S.10}$$

where the derivatives of the invariants are

$$\frac{\partial I_1}{\partial \mathbf{C}} = \frac{\partial tr(\mathbf{C})}{\partial \mathbf{C}} = \frac{\partial(\mathbf{I}:\mathbf{C})}{\partial \mathbf{C}} = \mathbf{I}, \tag{S.11}$$

$$\frac{\partial I_4}{\partial \mathbf{C}} = \boldsymbol{n} \otimes \boldsymbol{n}. \tag{S.12}$$

Combining (S.2)-(S.12), the explicit form of stress and electric field can be derived:

$$\mathbf{S} = C_1 \exp\left(\frac{I_1 - 3}{I_c}\right)\mathbf{I} + k\left(I_4 - 1\right)\boldsymbol{n} \otimes \boldsymbol{n} + \frac{1}{\varepsilon}\hat{\mathbf{D}} \otimes \hat{\mathbf{D}} - p\mathbf{C}^{-1}, \tag{S.13}$$

$$\boldsymbol{\sigma} = C_1 \exp\left(\frac{I_1 - 3}{I_c}\right)\mathbf{b} + k\left(I_4 - 1\right)\mathbf{F}\left(\boldsymbol{n} \otimes \boldsymbol{n}\right)\mathbf{F}^{\mathrm{T}} + \frac{1}{\varepsilon}\mathbf{F}\hat{\mathbf{D}} \otimes \mathbf{F}\hat{\mathbf{D}} - p\mathbf{I}, \tag{S.14}$$

$$\mathbf{P} = C_1 \exp\left(\frac{I_1 - 3}{I_c}\right)\mathbf{F} + k\left(I_4 - 1\right)\mathbf{F}\left(\boldsymbol{n} \otimes \boldsymbol{n}\right) + \frac{1}{\varepsilon}\mathbf{F}\left(\hat{\mathbf{D}} \otimes \hat{\mathbf{D}}\right) - p\mathbf{F}\mathbf{C}^{-1}, \tag{S.15}$$

$$\hat{\mathbf{E}} = \frac{1}{\varepsilon}J^{-1}\mathbf{C}\hat{\mathbf{D}}. \tag{S.16}$$

The developed model is first employed to describe the anisotropic mechanical response of shearing DLCEs by fitting with the uniaxial tension data sets, which are parallel and perpendicular to , respectively. Specifically, we set $\boldsymbol{n} = \left(1, 0, 0\right)$ for model fitting, as shown in Fig. S17.

For uniaxial tension parallel to , the nematic liquid crystals will not affect the other two directions, thus the deformation gradient can be represented as $\mathbf{F} = \lambda_1 \mathbf{e}_1 \otimes \mathbf{e}_1 + \frac{1}{\sqrt{\lambda_1}}\mathbf{e}_2 \otimes \mathbf{e}_2 + \frac{1}{\sqrt{\lambda_1}}\mathbf{e}_3 \otimes \mathbf{e}_3$, where λ_1 is stretch and $\mathbf{e}_i$ are the basis directions. Using the traction free boundary condition in $\mathbf{e}_2$ and $\mathbf{e}_3$ direction, the principal nominal stress can be obtained as

$$P_1 = C_1 \exp\left(\frac{I_1 - 3}{I_c}\right)\left(\lambda_1 - \frac{1}{\lambda_1^2}\right) + 2k\left(\lambda_1^2 - 1\right)\lambda_1, \tag{S.17}$$

$$P_2 = P_3 = 0, \tag{S.18}$$

where $I_1 = \lambda_1^2 + 2/\lambda_1$ and $I_2 = 2\lambda_1 + 1/\lambda_1^2$.

For uniaxial tension perpendicular to , the loading direction is set as $\left(0, 1, 0\right)$. The liquid crystals will limit the compression along the nematic direction, thus the other two principal stretch λ_1 and λ_2 will not equal to each other. Therefore, the deformation gradient should be written as $\mathbf{F} = \lambda_1 \mathbf{e}_1 \otimes \mathbf{e}_1 + \lambda_2 \mathbf{e}_2 \otimes \mathbf{e}_2 + \frac{1}{\lambda_1 \lambda_2}\mathbf{e}_3 \otimes \mathbf{e}_3$. With the traction free boundary condition in $\mathbf{e}_1$ and $\mathbf{e}_3$ direction, the mechanical equilibrium equations can be given from Eq. (S.15):

$$P_1 = C_1 \exp\left(\frac{I_1 - 3}{I_c}\right)\lambda_1 + 2k\left(\lambda_1^2 - 1\right)\lambda_1 - \frac{p}{\lambda_1} = 0, \tag{S.19}$$

$$P_2 = C_1 \exp\left(\frac{I_1 - 3}{I_c}\right)\lambda_2 - \frac{p}{\lambda_2}, \tag{S.20}$$

$$P_3 = C_1 \exp\left(\frac{I_1 - 3}{I_c}\right)\frac{1}{\lambda_1\lambda_2} - p\lambda_1\lambda_2 = 0. \tag{S.21}$$

where λ_1 and are two unknown independent variables, which can be solved by the two traction free conditions. Then the principal stress P_2 can be analytically calculated with λ_2 given by experimental data using (S.20). Using (S.17) and (S.20), we can fit the anisotropic mechanical behavior in two directions simultaneously. An iteration is performed by varying the values of mechanical parameters and to minimize the error between experimental data and analytical results defined as $\sum_i\left[\left(P_1^{exp} - P_1\right)^2 + \left(P_2^{exp} - P_2\right)^2\right]$, where i represents the total number of data points. Since the bending deformation relates relatively small stretch only, the model is fitted within a stretch smaller than 1.2, and the fitted stress-stretch curves are shown in Fig. S18.

After the mechanical parameters are obtained, the model is then applied to capture the electro-activated behavior under certain electric field in the thickness direction ($\boldsymbol{e}_3$). The existing of nematic liquid crystals differs the electro-mechanical behavior in the vertical plane, thus the deformation gradient is still $\mathbf{F} = \lambda_1\mathbf{e}_1 \otimes \mathbf{e}_1 + \lambda_2\mathbf{e}_2 \otimes \mathbf{e}_2 + 1/(\lambda_1\lambda_2)\,\mathbf{e}_3 \otimes \mathbf{e}_3$. With a free boundary condition and (S.15), the equilibrium equations can be written as

$$P_1 = C_1 \exp\left(\frac{I_1 - 3}{I_c}\right)\lambda_1 + 2k\left(\lambda_1^2 - 1\right)\lambda_1 - \frac{p}{\lambda_1} = 0, \tag{S.22}$$

$$P_2 = C_1 \exp\left(\frac{I_1 - 3}{I_c}\right)\lambda_2 - \frac{p}{\lambda_2} = 0, \tag{S.23}$$

$$P_3 = C_1 \exp\left(\frac{I_1 - 3}{I_c}\right)\frac{1}{\lambda_1\lambda_2} - p\lambda_1\lambda_2 + \varepsilon\tilde{E}^2\lambda_1^3\lambda_2^3 = 0. \tag{S.24}$$

Similarly, three independent variables λ_1, λ_2 and can be solved with the given applied nominal electric field $\tilde{E}$ from experimental data. Additionally, the in-plane deformation perpendicular to (direction $\mathbf{e}_2$) contributes mainly to the electro-mechanical behavior. Thus, we fit with the data in direction 2 to obtain the relative dielectric permittivity , as shown in Fig. S19. The model parameters fitted are $C_1 = 0.079$ MPa, $I_c = 1.01$, $k = 1.08$ MPa, $\varepsilon_r = 4.9$. Then the model is implemented in COMSOL Multiphysics. Using one-element simulation, the FEA program is validated by comparing with the theoretical electro-mechanical responses, as shown in Fig. S21A and Fig.S20. The results also demonstrate that our finite element simulation can capture the anisotropic electro-activated behavior (Fig. S21B). During the FEA simulation, a quasi-incompressible model is used instead to avoid

the non-convergence issues caused by additional degrees of freedom due to incompressible conditions. The volumetric free energy is represented by a second order function of J, written as $\frac{\kappa}{2}(J-1)^2$. The bulk modulus κ is set to 200 times the shear modulus C_1 to simulate the nearly incompressible condition. As for the isotropic non-sheared DLCE, a linear model is adopted as its constitutive model, for the electric actuated deformation of the isotropic DLCE is indeed very small—negligible for practical purposes—and can be considered to remain within the linear elastic regime. The Young's modulus Y of isotropic DLCE is fitted with experimental data as 0.5338 MPa, and the Poisson's ratio ν set as 0.49 to consider the quasi-incompressible condition of the elastomer. The fitting process of the dielectric permittivity of non-sheared DLCE can be derived as follows. Following the general Hooke's law, the principal strain can be given as:

$$\varepsilon_i^{elastic} = \frac{1+\nu}{Y}\sigma_i - \frac{\nu}{Y}\sigma_v, \tag{S.25}$$

where $\varepsilon_i^{elastic}$ is the principal strain, σ_i is the principal stress and σ_v is the volumetric stress. Here, the volumetric stress is the Maxwell stress raising from the dielectric part, one can derive the stress using the dielectric part in our anisotropic model as

$$\sigma_v = \frac{1}{2}\varepsilon E^2, \tag{S.26}$$

where E is the true electric field strength with $E = \frac{\phi}{H\lambda_3}$, ϕ is the electric potential on the surface. With this in hand, the principal strain $\varepsilon_3^{elastic}$ can be expressed as

$$\varepsilon_3^{elastic} = -\frac{1+2\nu}{Y}\frac{\varepsilon E^2}{2}. \tag{S.27}$$

Substituting into the identities of true electric field strength $E - \frac{\phi}{H\lambda_3} = 0$, and using the relation between strain and stretch ratio $\lambda_3 = e^{\varepsilon_3}$, we have

$$Ee^{-\frac{(1+2\nu)\varepsilon E^2}{2Y}} - \frac{\phi}{H} = 0. \tag{S.28}$$

The true electric field strength and then strain can be computed by solving this equation, ultimately the dielectric permittivity can be calibrated as $\varepsilon_r = 2.98$, as shown in Fig.S18B.

As for the boundary conditions for different fields, according to the different situations of each demo, the mechanical boundary conditions are equivalent to those in the experiment, respectively. As for the electrical part, we adopt the weak form PDEs Interface in COMSOL to solve the changing electric field during actuation. The weak form of the electric field can be written as

$$\int \mathbf{F}^{-1}\nabla_{\mathbf{X}}\phi \cdot \mathbf{F}^{-1}\nabla_{\mathbf{X}} w dV = 0, \tag{S.29}$$

where w is the weighting function. The derivation of the weak form for the electric part can refer to our previous work (*48*). The boundary condition of the electric field adopts the Dirichlet boundary condition in COMSOL to assign the applied electric potential to be the same as that in the experimental setup. Hence, during simulation, we set the potential as 0 on the surface of the negative electrode and a value for the actual potential on the surface of the positive electrode. The quadratic serendipity shape function is chosen for the element to ensure accuracy. Meanwhile, we chose the hexahedral element partitioning geometries of every demo in our work. The meshes are generated by sweeping the generated free quadrilateral mesh from one surface to another surface of the geometry in COMSOL. We show the generated meshes of four representative actuators in Fig. S24.

Supplementary Note 3 Inverse design method: mapping transport method

In this work, we demonstrate inverse design using a target panda face as an example. The inverse design here is to provide a guidance according to target shapes for manufacture of DLCE actuators, where it provides the shearing location and the shear direction at the same time.

First, the 3D target shape is digitized into coordinate data using a line laser profile sensor (UC3D230ED-800X600-R-UC3D). The scanned data are discretized into a mesh grid via MATLAB's *meshgrid* function, yielding the target coordinates (Fig. 4a(ii) and Fig. S31). Since the initial DLCE actuator is a flat plane which can be expressed as $(X, Y, Z=0)$, the inverse design should map the material points $(X, Y, Z=0)$ on the initial flat plane into the corresponding points in the target points (x, y, z). Given the fixed boundary conditions of the initially flat structure, in-plane deformations in the X-Y plane are negligible, permitting the assumptions $x = X$ and $y = Y$, and thus the out-of-plane displacement is described by $z = z(X, Y)$. Such assumption enforces zero length change along contour lines $z(X, Y)$ (also along the direction $\mathbf{e}_1$), a constraint imposed by nematic liquid crystal confinement. However, length changes along the gradient vector $\nabla z(X, Y)$ (also along the direction $\mathbf{e}_2$), remain permissible. In other word, the stretch $\lambda_1 \approx 1$ holds due to the nematic liquid crystals, and the pattern of the distributions of liquid crystal mesogens must be the same as contour lines *(37)* $z = z(X, Y)$. Thereby, the next step is to discretize the continuous contour lines $z = z(X, Y)$ into separated segments with specific directions. Specifically, for each height level of the contour lines, the corresponding closed contour line is extracted, and then these contour lines are discretized into several segments whose number is controlled by needs. With the separated segments, we calculate the angles between each segment and the horizontal x axis. Since we choose 0°, 60°, and 120° as the shear directions for monodomain DLCE, segments are assigned to the nearest of three predefined shear directions (0°, 60°, or 120°) for monodomain DLCE alignment. This discretization determines the nematic director field governing the DLCE actuation. The original separated segments and assigned segments are shown in Fig. S32.

After orienting the segment, we need to design the distribution of monodomain DLCE, which is calculated from the locally desired deformation. The local required deformation (stretch) λ_2 is derived from the surface area change, quantified via the first fundamental form of the surface. The relative change in surface is decided by:

$$\frac{da}{dA} = \frac{da}{dXdY} = \sqrt{EG - F^2}\,, \tag{S.30}$$

where $E = \frac{\partial(x,y,z)}{\partial X}\frac{\partial(x,y,z)}{\partial X}$, $F = \frac{\partial(x,y,z)}{\partial X}\frac{\partial(x,y,z)}{\partial Y}$ and $G = \frac{\partial(x,y,z)}{\partial Y}\frac{\partial(x,y,z)}{\partial Y}$, are the basic quantity representing the change of the differential geometry element along the direction of the basis vector and angle. The deformation assumption will lead

$E = \frac{\partial z}{\partial X}\frac{\partial z}{\partial X}$, $F = \frac{\partial z}{\partial X}\frac{\partial z}{\partial Y}$ and $G = \frac{\partial z}{\partial Y}\frac{\partial z}{\partial Y}$. At the same time, since $\lambda_1 \approx 1$, the relative change in area is

$$\frac{da}{dXdY} = \lambda_1\lambda_2 \approx \lambda_2 . \tag{S.31}$$

Therefore, combining (S.30) and (S.31) yields

$$\lambda_2 = \sqrt{\left|\nabla z(X,Y)\right|^2 + 1} . \tag{S.32}$$

Considering the multilayer thin DLCEs in this work (the ratio between thickness and length is smaller than 1/20), the bending stiffness and moments can be ignored. Thus, with the developed model, the locally actuated stretch λ_2 can be solved by substituting $\lambda_1 \approx 1$ into equations (S.22)-(S.24):

$$\exp\left(\frac{I_1 - 3}{I_3}\right)\left(1 - \lambda_2^{-4}\right) = \hat{\phi} . \tag{S.33}$$

Here we have $I_1 = 1 + \lambda_2^2 + \frac{1}{\lambda_2^2}$, and $\hat{\phi}$ denotes the nondimensionalized electric potential which can be controlled with the number of shearing layers N_m as

$$\hat{\phi} = \frac{N_m}{N}\hat{\phi}_{\max} , \tag{S.34}$$

where N denotes the total number of layers (we choose 6 here) and $\hat{\phi}_{max} = \sqrt{\frac{\varepsilon}{C1}}\frac{\phi}{t_0}$, ϕ is the required voltage and t_0 is the initial thickness of each elastomer layer. Therefore, once the locally required stretch λ_2 is calculated, substituting (S.34) into (S.33), the local number of shearing layers can be determined using (S.33)-(S.34) as

$$N_m = \frac{N}{\hat{\phi}_{\max}}\sqrt{\exp\left(\frac{I_1 - 3}{I_c}\right)\left(1 - \lambda_2^{-4}\right)} . \tag{S.35}$$

Since the number of shearing layers must be an integer, N_m is rounded to the nearest integer after being calculated (Fig. S35-S38). Using the local number of shearing layers and overlaying the figure of nematic field directors in Fig. S32B, we can obtain the pattern of monodomain DLCE of each layer, as shown in Fig. S33.

By using the Bresenham algorithm to identify the grids where oriented directors (blue segments) coincide with the monodomain area (red areas), each pattern in Fig. S33 can be further discretized into shear patterns with specific three directions. The discretized shear

patterns with different colors are listed in Fig. S37 according to layer index where red denotes 0° shearing direction, green denotes 60° shearing direction and blue denotes 120° shearing direction. In summary, we integrate the above steps into program flowchart in Fig. S34. Using the same program, the shearing patterns for the other two complex target shape Graptoveria Amethorum and Yellow River landform are listed in Fig. S37 and Fig. S38, respectively.

Supplementary Note 4 Materials and methods

Experimental design

This study aims to validate the hypothesis that shear force-induced molecular orientation can effectively align liquid crystal mesogenic units in main-chain DLCE systems during layer-by-layer digital light processing (DLP) printing without requiring pre-alignment treatments. To evaluate the efficacy of this fabrication approach, we designed, developed, and demonstrated a series of DLP-printed shape-morphing actuators capable of achieving complex deformations, including zero, positive, and negative Gaussian curvature configurations. Furthermore, leveraging inverse design strategies, we fabricated sophisticated topographical structures and successfully demonstrated their application in liquid transport.

Materials

Diacrylate liquid crystal (LC) monomers RM257 and RM82 were sourced from Wilshire Technologies. EDDT, TATATO, TPO, the radical inhibitor butylated hydroxytoluene (BHT), and anhydrous tetrahydrofuran (THF) were procured from Sigma-Aldrich. All chemical reagents and solvents were utilized in their as-received state without additional purification steps.

Characterizations

Fourier transform infrared (FT-IR) spectroscopy was performed with an FT-IR Spectrum Two (PerkinElmer) spectrometer in the range of 400-4000 cm^{-1}. UV absorption spectra were performed with a UV 2700 (Shimadzu) in the range of 190-900 nm. Alignment of printed DLCE samples was characterized by X-ray scattering measurements on a SAXS/WAXS XEUSS 3.0 system with an excillum METALJET microfocus X-ray source (λ =0.135 nm) with a sample to detector (EIGER2 Si 1M, Dectris) distance of 42.5 mm for 600 s to capture the mesogens-mesogen correlations at q ~ 1.5 $Å^{-1}$. Wide-angle X-ray scattering samples comprised four-layer printed DLCE unidirectional strips (200μm thick). The nematic orientation parameter was characterized by Hermans' orientation parameter (S), given as

$$cos^2\varphi = \frac{\int_0^{\pi} I(\varphi) cos^2\varphi sin\varphi d\varphi}{\int_0^{\pi} I(\varphi) sin\varphi d\varphi}, \quad \text{(S.31)}$$

$$S = \frac{3cos^2\varphi - 1}{2}, \quad \text{(S.32)}$$

where $\langle \cos^2 \phi \rangle$ is the average cosine square of the angles between the long axis of individual mesogens and the global LC director. $I(\phi)$ is the angle-dependent scattering intensity from the WAXS patterns. Orientation parameter calculations were performed using Matlab (Mathworks, Natick, MA).

Gel experiment

The sample was weighed to its original mass and immersed in the tetrahydrofuran solution for 48h. The final mass is weighed after drying in a vacuum oven at 50°C for 24 hours. The ratio of original mass to final mass is the gel fraction.

Tensile test

The tensile tests were conducted on a tensile machine (UTM4203, Suns Technology Stock Co., Ltd.). In the uniaxial tensile test, the polymer film was cut into a dumbbell shape with a neck width of 2 mm wide and a gauge length of 12 mm. The specimens were stretched at a constant stretch rate of 20 mm min^{-1} until they broke.

Measurement of dielectric properties

Dielectric properties in the range of 10^2 Hz to 10^6 Hz were characterized by a dielectric spectroscopy (Keysight 4980L, PolyK). The films were sputtered with silver electrodes for 60 s (KT-1650PVD).

DLCE resin rheology

Rheological properties of NAT-LCE inks were measured by a rotational rheometer (Physical MCR 302, Anton Paar, Austria), and a 25 mm steel Peltier plate was used with a 0.5 mm gap distance. The shear rate was swept from 10^{-2} to 10^2 s^{-1} during the viscosity measurement.

Preparation of DLCE liquid resin

To initiate the thiol-acrylate/thiol-ene click reaction, EDDT, RM257, RM82, and TATATO were dissolved in anhydrous tetrahydrofuran (THF) at controlled molar ratios within amber glass vials. The solution was then supplemented with 1 wt% TPO (a photoinitiator) and 2 wt% butylated hydroxytoluene (BHT) to mitigate premature thermal polymerization. The reaction mixture was continuously stirred at 200 rpm on a heating plate maintained at 65 °C for 4 hours to ensure complete oligomerization.

DLP of DLCE actuators

All DLP-compatible design files were initially created using the CAD software SolidWorks. These files were subsequently imported into Autodesk Print Studio, where they were sliced into photo-pattern layers and converted into the ".ctb" format prior to being transmitted to the Autodesk Ember printer for fabrication. The oligomerized liquid resin was transferred to the resin vat and allowed to settle for 5 minutes to minimize air entrapment before initiating the printing process.

For thin structures (thickness (h) less than 50 μm, fewer than 10 layers), the printer's in-plane (xy) resolution was measured at 150 μm, achieved using a 1280 × 800 pixel UV projector with a maximum build area of 70 mm × 40 mm. For thicker structures (50 μm < h < 200 μm, 10 to 40 layers), the resolution was adjusted to a range of 150 to 300 μm because of stronger light scattering to ensure optimal layer adhesion and structural integrity. The axial (z-axis) resolution of the printer was maintained at 50 μm throughout the fabrication process. Following printing, all specimens underwent a two-stage post-curing treatment: (1) bilateral UV exposure (λ = 365 nm, intensity = 25 mW cm^{-2}, 9 s per side) to ensure complete

crosslinking, followed by (2) thermal annealing at 70°C under vacuum for 24 hours to eliminate residual solvents and optimize material dielectric properties.

Preparation of SWCNT electrodes

8 mg SWCNT and 1.8 g SDS were mixed in 180 g deionized water and then ultrasonicated (400 W of power) for 30 min. SWCNT dispersions were obtained from the supernatant after centrifugation and decantation. The dispersion solution was then suction filtered through a PVDF microporous membrane with 0.22 μm^{-1} pore diameter to form a SWCNT layer on the membrane ready for the transfer printing later. The transmittance of the electrode at 530 nm was 85.7%T.

Figures and Tables

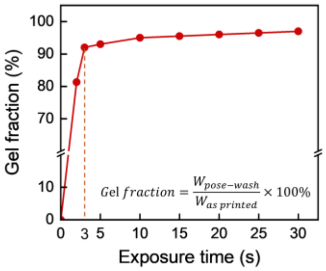

Supplementary Fig. 1. Gel content measured upon different UV exposure durations.

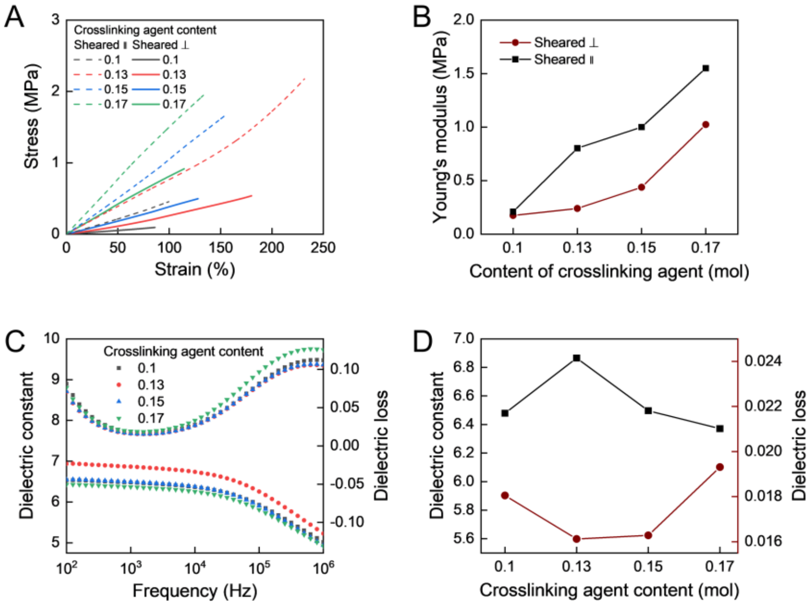

Supplementary Fig. 2 Effect of crosslinker content on the dielectric properties of DLCEs. (A), Tensile stress-strain curves parallel and perpendicular to the liquid crystal alignment direction under different crosslinker contents. **(B)**, Modulus variation parallel and perpendicular to the liquid crystal alignment direction with varying crosslinker contents. **(C)**, Frequency-dependent dielectric constant and dielectric loss at different crosslinker contents. **(D)**, Evolution trends of dielectric constant and dielectric loss with different crosslinker contents.

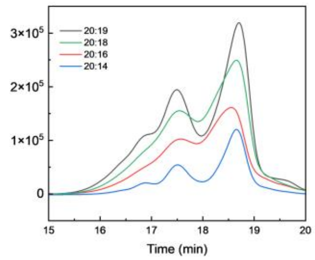

Supplementary Fig. 3. Variation of liquid crystal prepolymer molecular weight with changing thiol-to-acrylate group ratios.

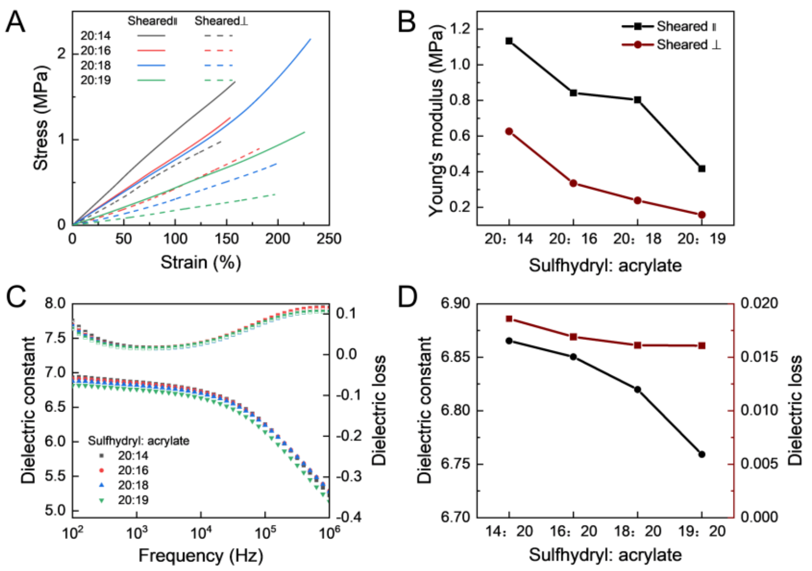

Supplementary Fig. 4 Effect of molecular weight on dielectric and mechanical properties of DLCEs. (A), Tensile stress-strain curves parallel and perpendicular to the LC alignment direction with different molecular weights. **(B)**, Modulus variation parallel and perpendicular to the LC alignment direction with different molecular weights. **(C)**, Frequency-dependent dielectric constant and dielectric loss with different molecular weights. **(D)**, Evolution trends of dielectric constant and dielectric loss with varying molecular weights.

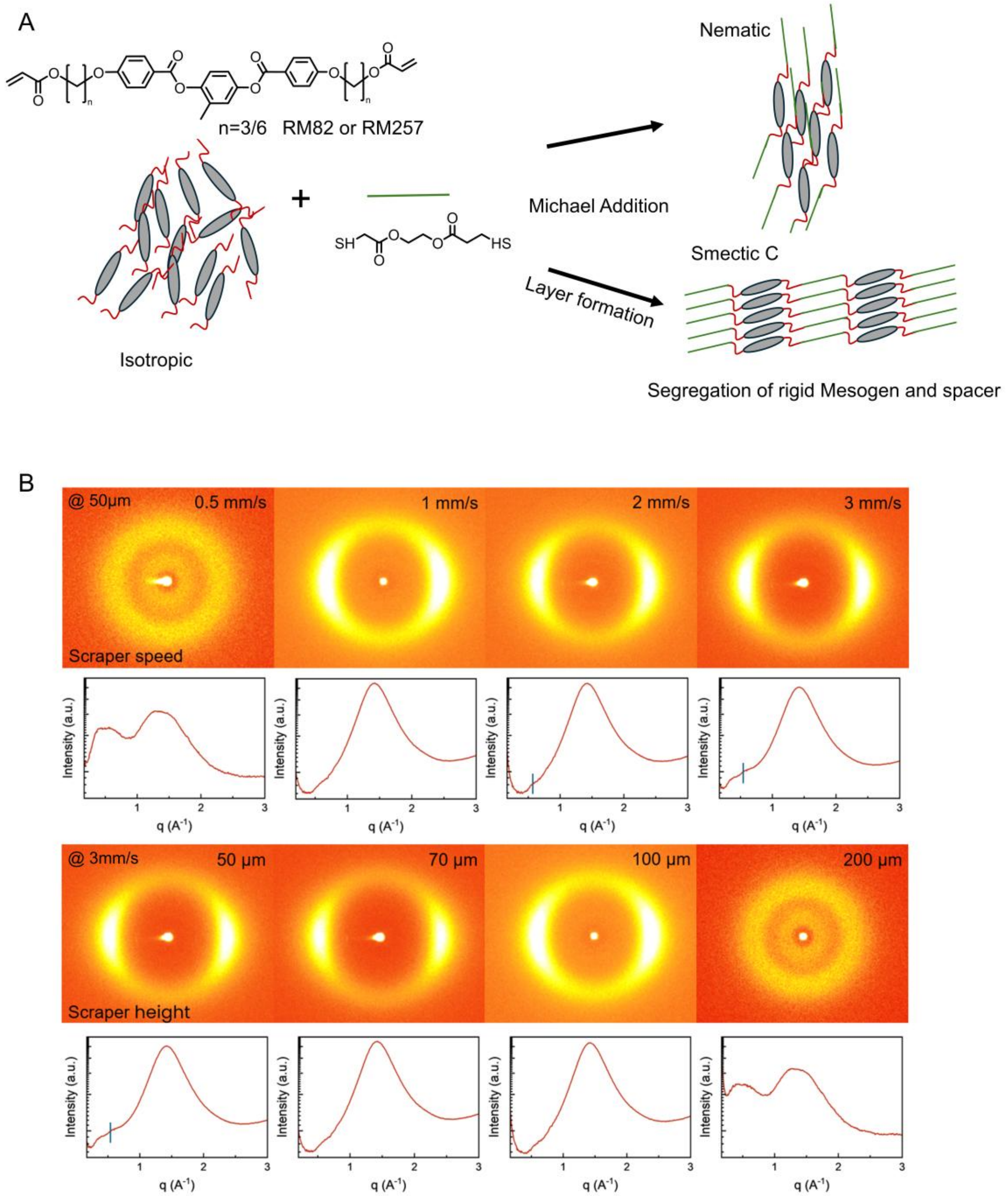

Supplementary Fig. 5 Crystalline phase of DLCE samples. (A), Nematic vs. smectic C phase alignment. **(B)**, 2D-WAXS patterns and azimuthal intensity distributions of printed DLCEs.

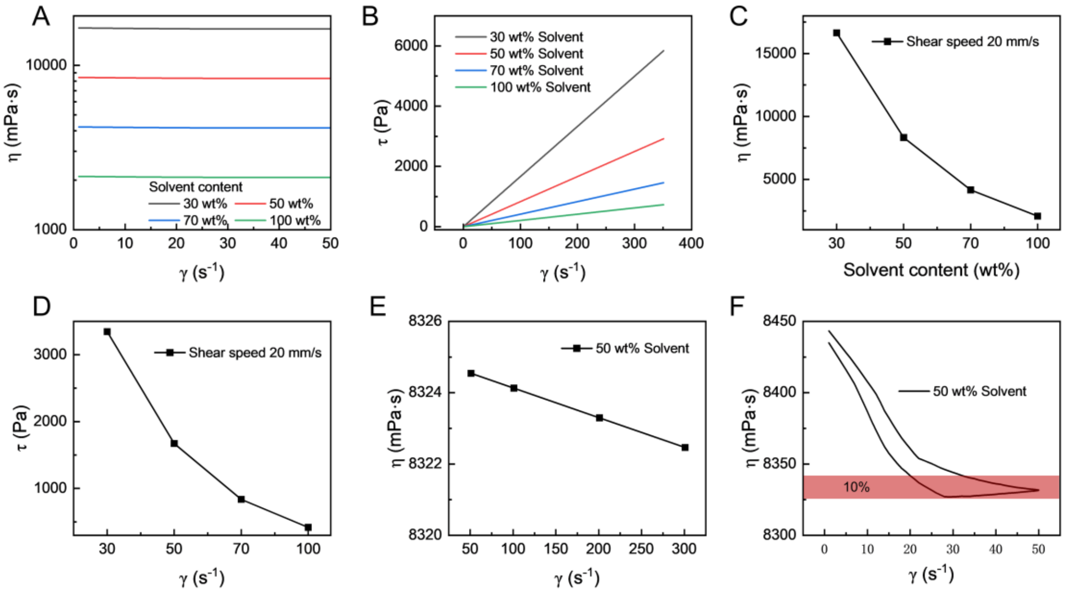

Supplementary Fig. 6 Rheological properties of DLCE prepolymer solutions. (A), Viscosity of inks with different solvent contents as a function of shear rate. **(B)**, Shear stress curves of inks with different solvent contents as a function of shear rate. **(C)**, Variation in viscosity with shear rate for inks with different solvent contents. **(D)**, Variation in shear stress with shear rate for inks with different solvent contents. **(E)**, Change in viscosity with increasing shear rate for ink containing 50% solvent. **(F)**, Rheological behavior of printable ink with 50% solvent.

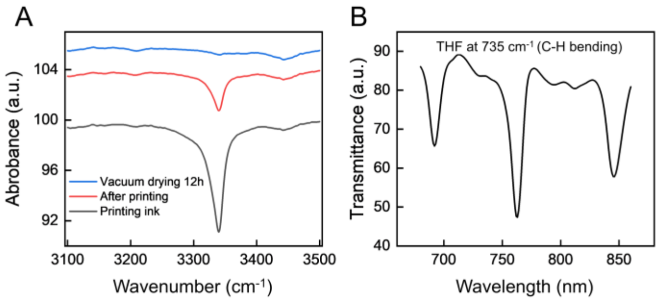

Supplementary Fig. 7 Optical and chemical characterization of DLCE printing materials. (A), FTIR spectra of the as-printed sample. **(B)**, UV-Vis spectra of the printing ink, as-printed sample, and after 12-hour vacuum oven drying.

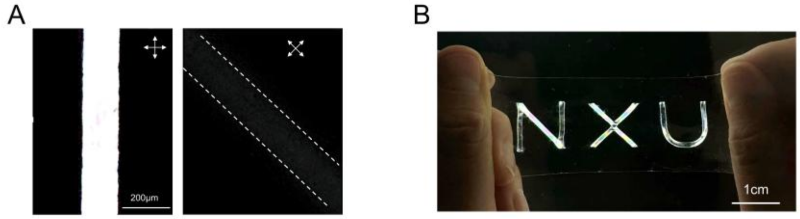

Supplementary Fig. 8 Characterization of anisotropic alignment in liquid crystal molecules. (A), POM images of printed DLCE showing birefringence caused by shear-induced alignment. **(B)**, Optical images of stretchable samples between 0° and 90° polarizers (scale bars, 1 cm).

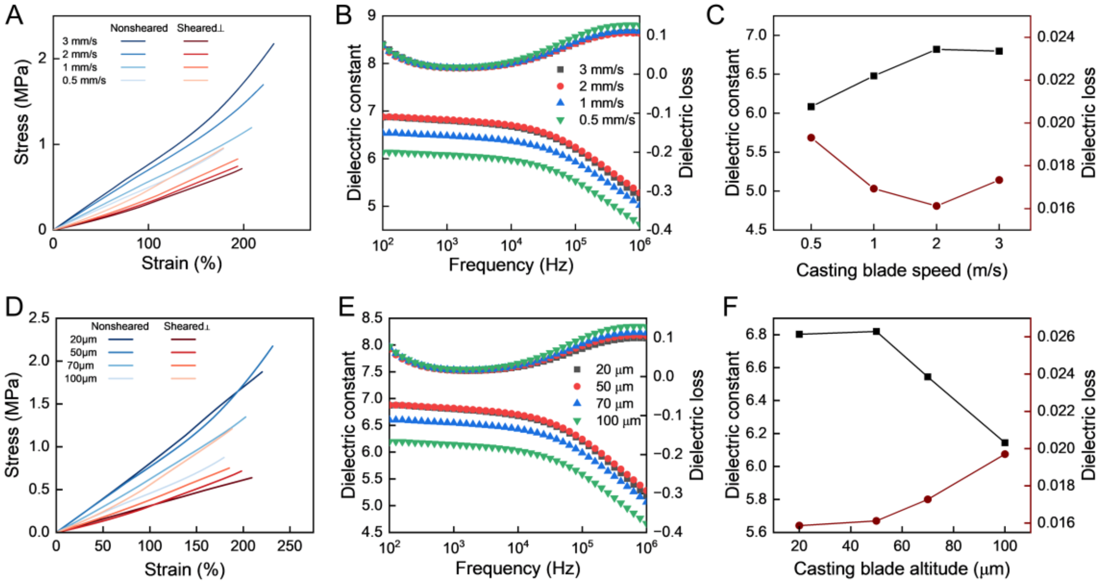

Supplementary Fig. 9 Printing-parameter-dependent mechanical and dielectric properties of DLCEs. (A), Tensile stress-strain curves of DLCE samples printed at different blade speeds. **(B)**, Dielectric constant and dielectric loss vs. frequency for DLCE samples printed at different blade speeds. **(C)**, Dielectric constant and dielectric loss at 1000 Hz for DLCE samples printed at different blade speeds. **(D)**, Tensile stress-strain curves of DLCE samples printed at different blade altitudes. **(E)**, Dielectric constant and dielectric loss vs. frequency for DLCE samples printed at different blade altitudes. **(F)**, Dielectric constant and dielectric loss at 1000 Hz for DLCE samples printed at different blade altitudes.

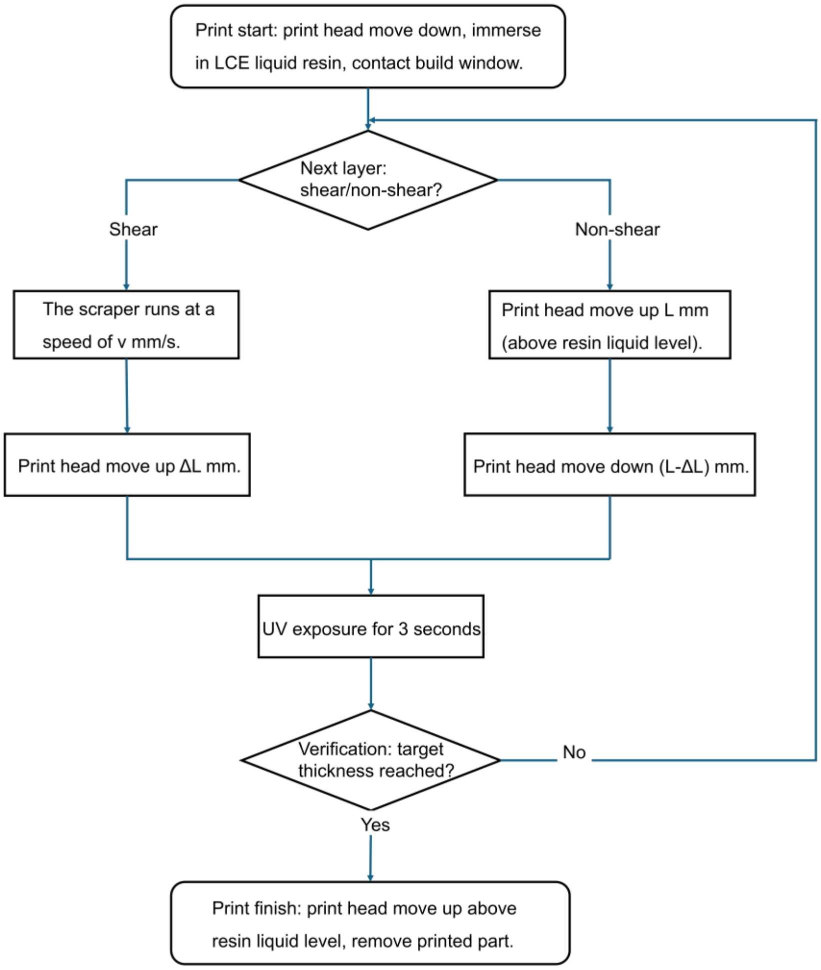

Supplementary Fig. 10 Additive manufacturing protocol for multi-layer compliant bending drives. (L as initial drop height, ΔL as single-layer height, v as blade speed.)

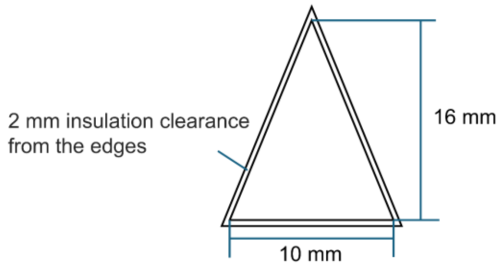

Supplementary Fig. 11 Geometry of the triangular DLCE actuator.

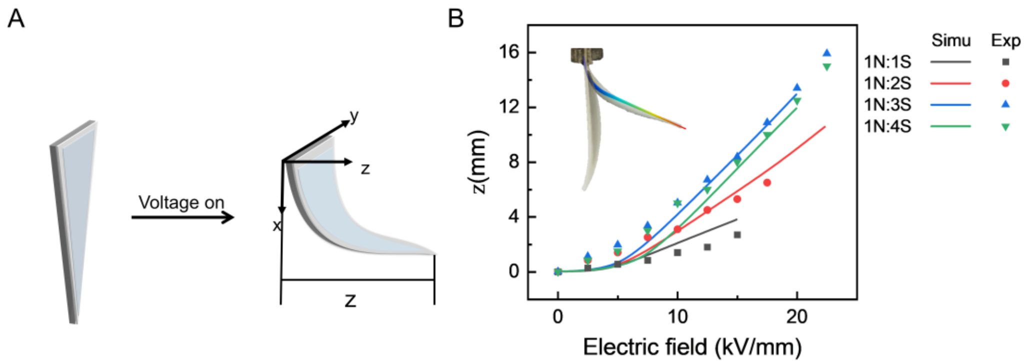

Supplementary Fig. 12 Comparison of experimental and simulation results for bending actuators. (A), Schematic diagram of a triangular actuator configuration and its displacement measurement. (**B),** Displacement variation of several actuator configurations under different electric fields (N: Nonsheared; S: Sheared).

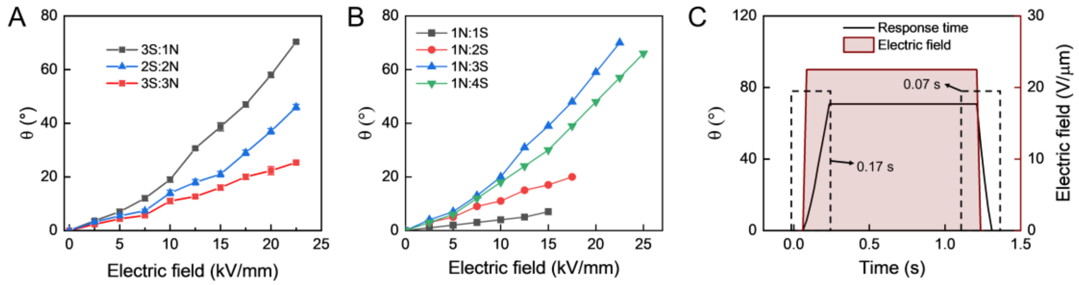

Supplementary Fig. 13 Actuation performance of triangular DLCE actuators. (A), Bending angles of actuators with different sheared (S)/nonsheared (N) layer combinations under varying electric fields (y-axis: angular displacement; x-axis: applied electric field). **(B)**, Bending angle versus electric field for actuators with a fixed P layer and varying numbers of M layers. **(C)**, Response and recovery time of the dielectric DLCE bending actuator.

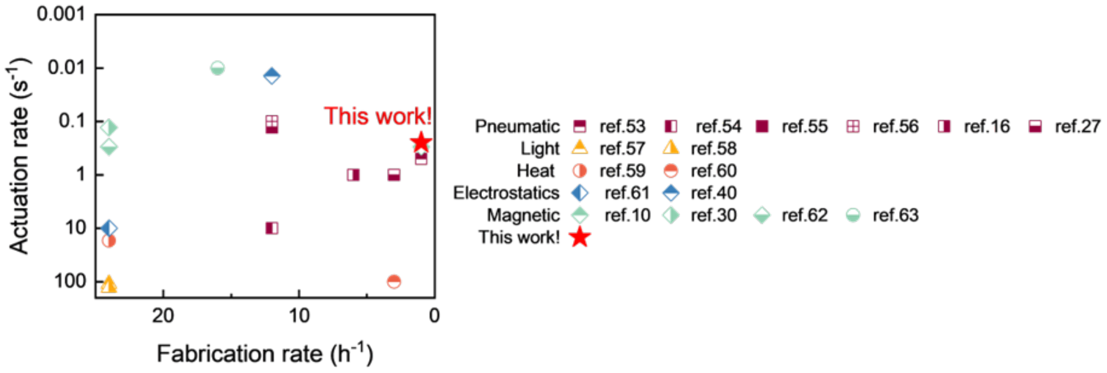

Supplementary Fig. 14 Actuation rate comparison between this work and other stimuli-responsive shape-morphing actuators (*10, 16, 27, 30, 40, 53-63*).

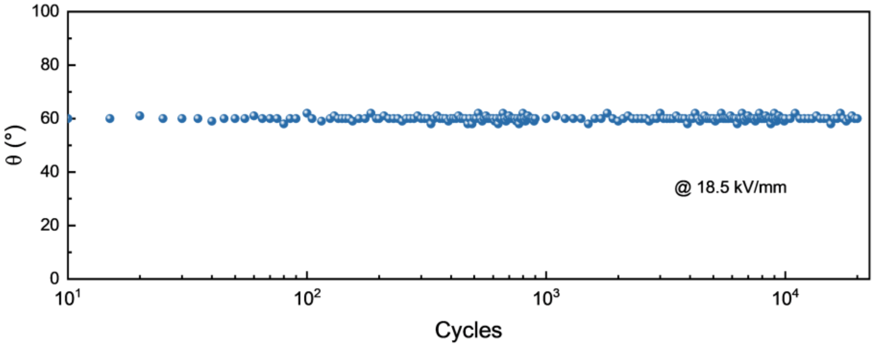

Supplementary Fig. 15 Cyclic loading test of the bending actuator.

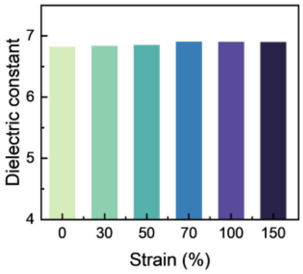

Supplementary Fig. 16 Dielectric constant of shearing DLCEs with different strains.

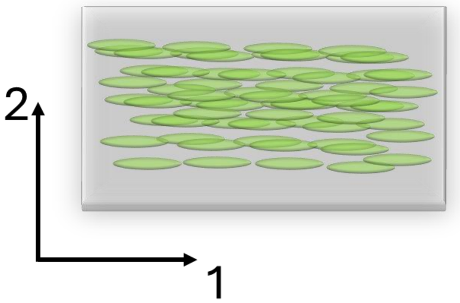

Supplementary Fig. 17 Nematic direction diagram.

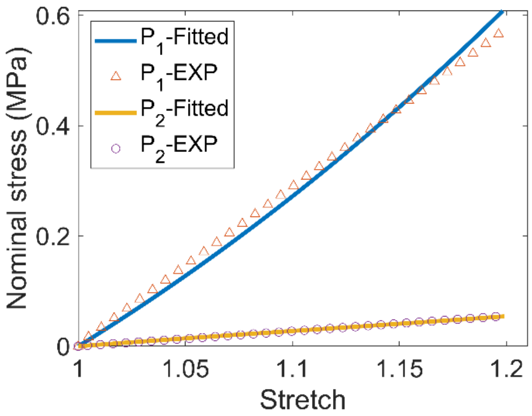

Supplementary Fig. 18 Anisotropic mechanical responses of DLCEs.

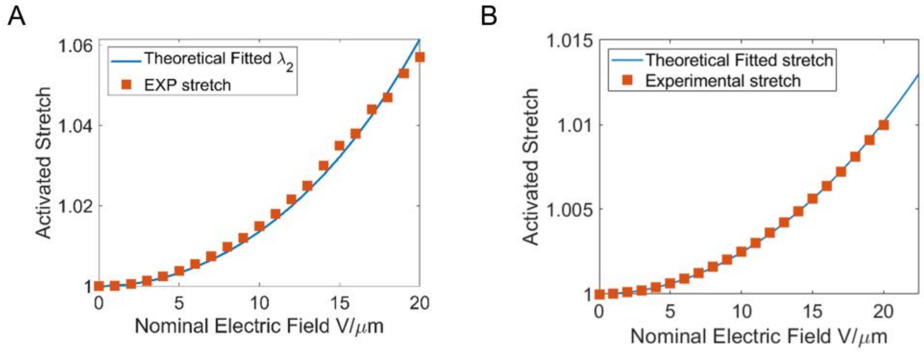

Supplementary Fig. 19 Calibration results of dielectric part DLCEs. (A) Sheared DLCE, the calibrated relative dielectric permittivity ε_r is 4.9. **(B)** Non-sheared DLCE with calibrated relative dielectric permittivity of 2.98.

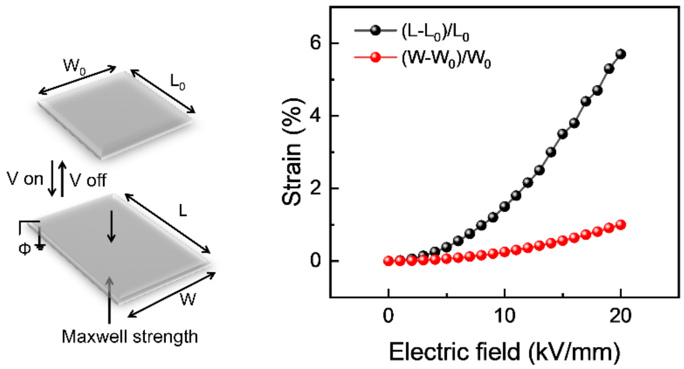

Supplementary Fig. 20 Deformation of DLCE along the direction parallel (W) and perpendicular (L) to the liquid crystal alignment under different electric fields, measured from a specimen under free boundary condition.

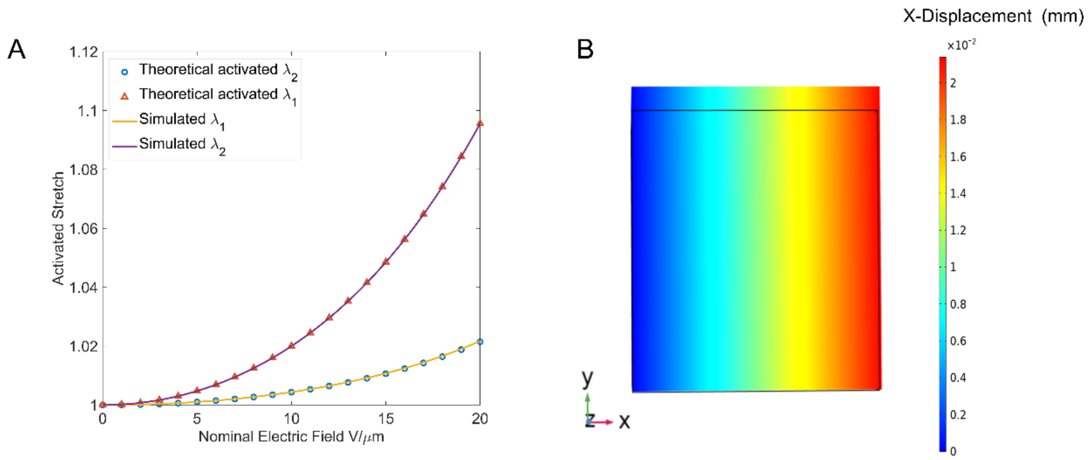

Supplementary Fig. 21 Simulation results for electro-mechanical behavior of DLCEs. (A), Comparison between theoretical and simulation results. **(B)**, One square element simulation for DLCEs, the solid line in black denotes the initial configuration of the DLCE element. The electro-stimulated deformation in the x-direction is relatively larger than in the y-direction.

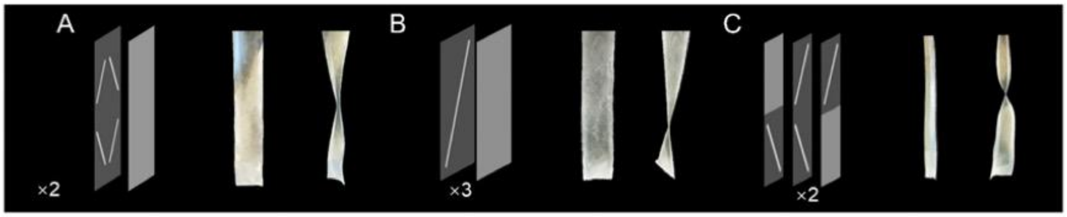

Supplementary Fig. 22 Shape-morphing of a rectangular DLCE actuator with complex configurations (the superscript ×2 indicates a bilayer stacked configuration). (A), Configuration 1. **(B)**, Configuration 2. **(C)**, Configuration 3.

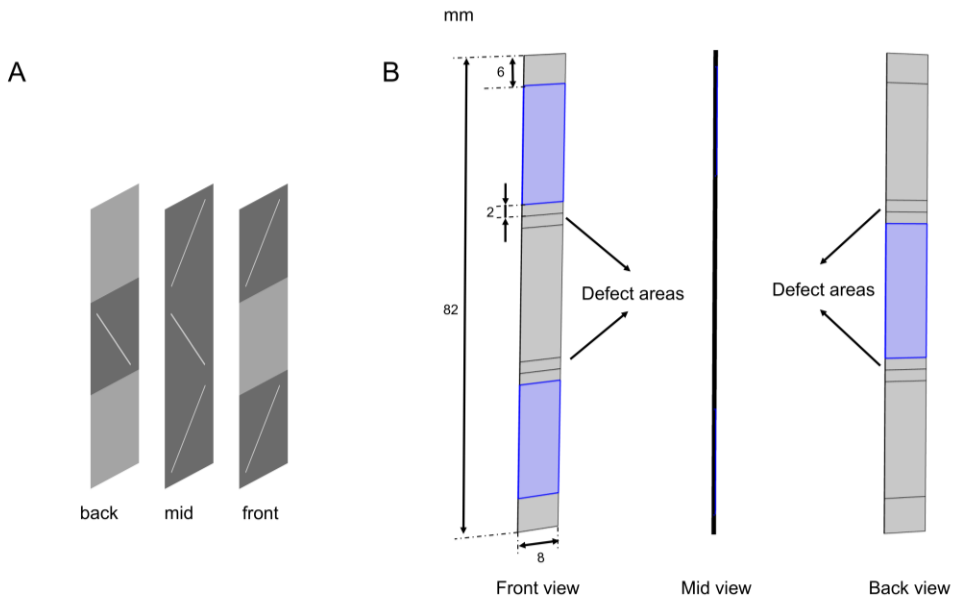

Supplementary Fig. 23 Evaluated active areas for the finite element simulation. (A), Configuration of electrodes and nematic directors. **(B)**, Front view and back view for the geometry of twisting actuator in the finite element simulation. Areas in blue denote the shearing areas while those in gray denote the nonsheared areas. The defect areas are set for the counteraction between adjacent shearing areas.

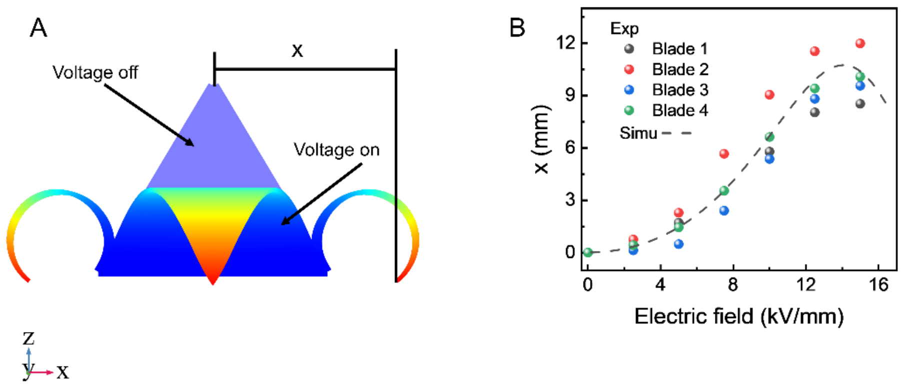

Supplementary Fig. 24 Comparison of experimental and simulation results for pyramid-shaped actuators. (A), Schematic diagram of the pyramid-shaped actuator configuration and its displacement measurement method. **(B)**, Displacement variation of the pyramid-shaped actuator under different applied electric fields.

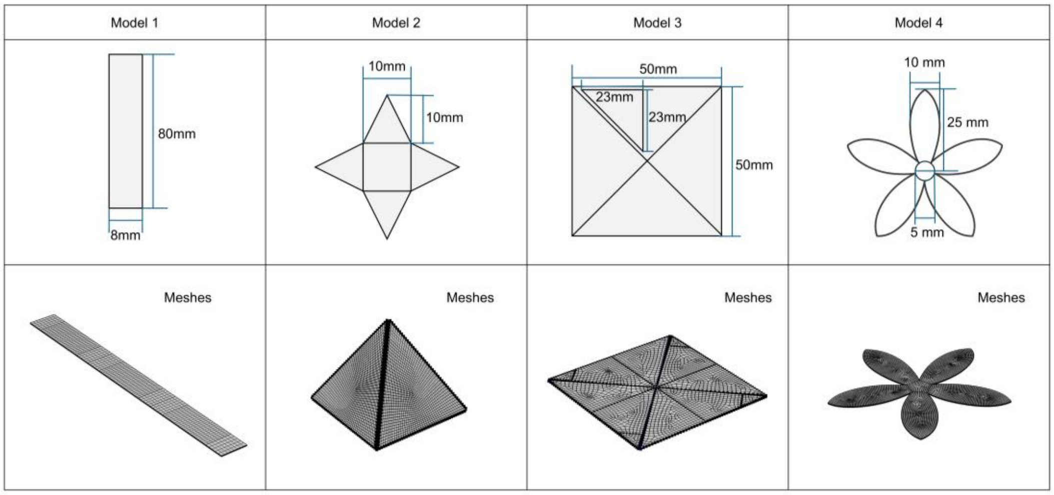

Supplementary Fig. 25 Programmable geometric states and meshes of DLCE actuators.

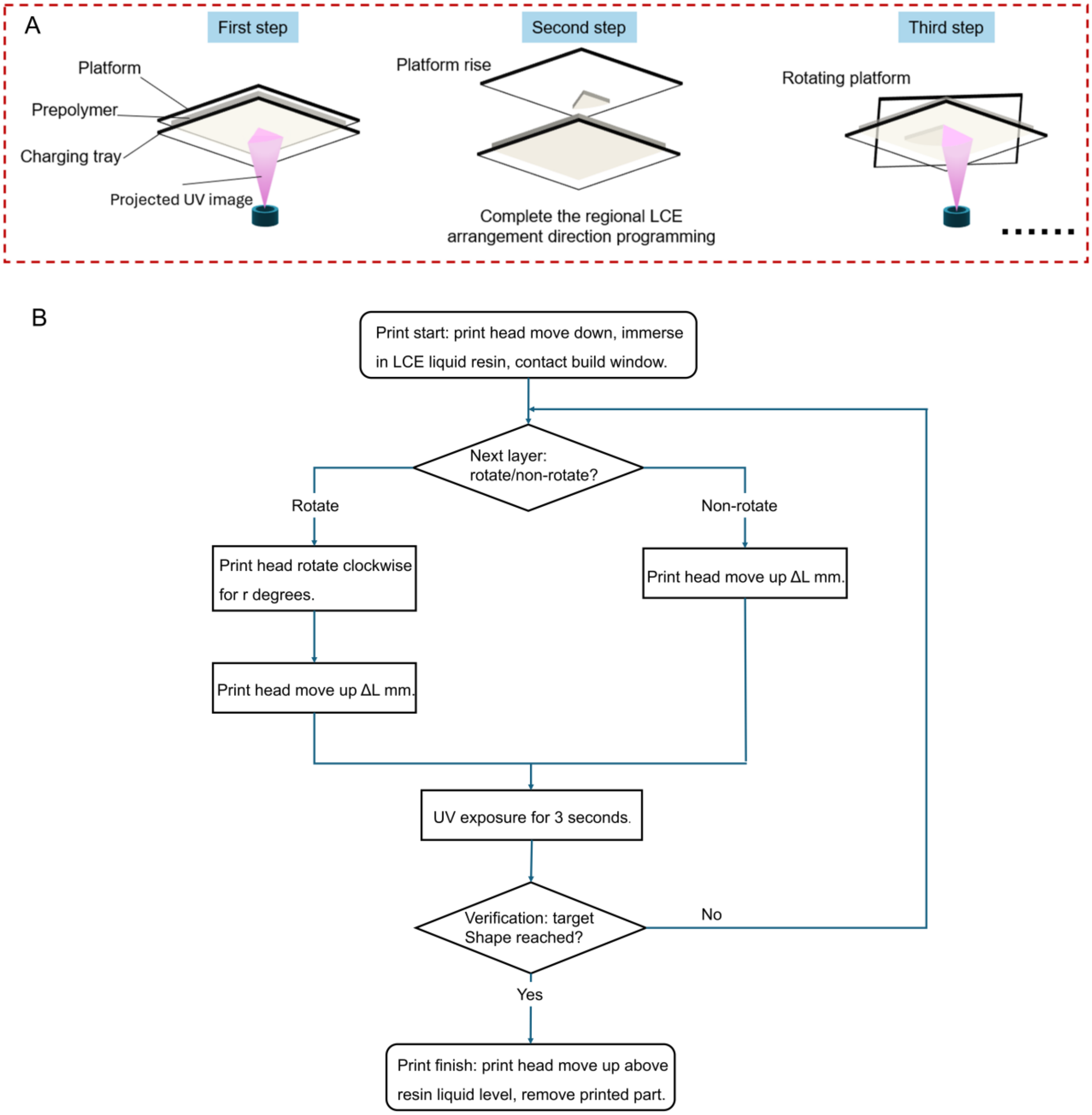

Supplementary Fig. 26 Platform operation process of 4D printing. (A), schematic and **(B)**, control program for circular actuators. (L as initial drop height, ΔL as single-layer height, r as platform rotation angle.)

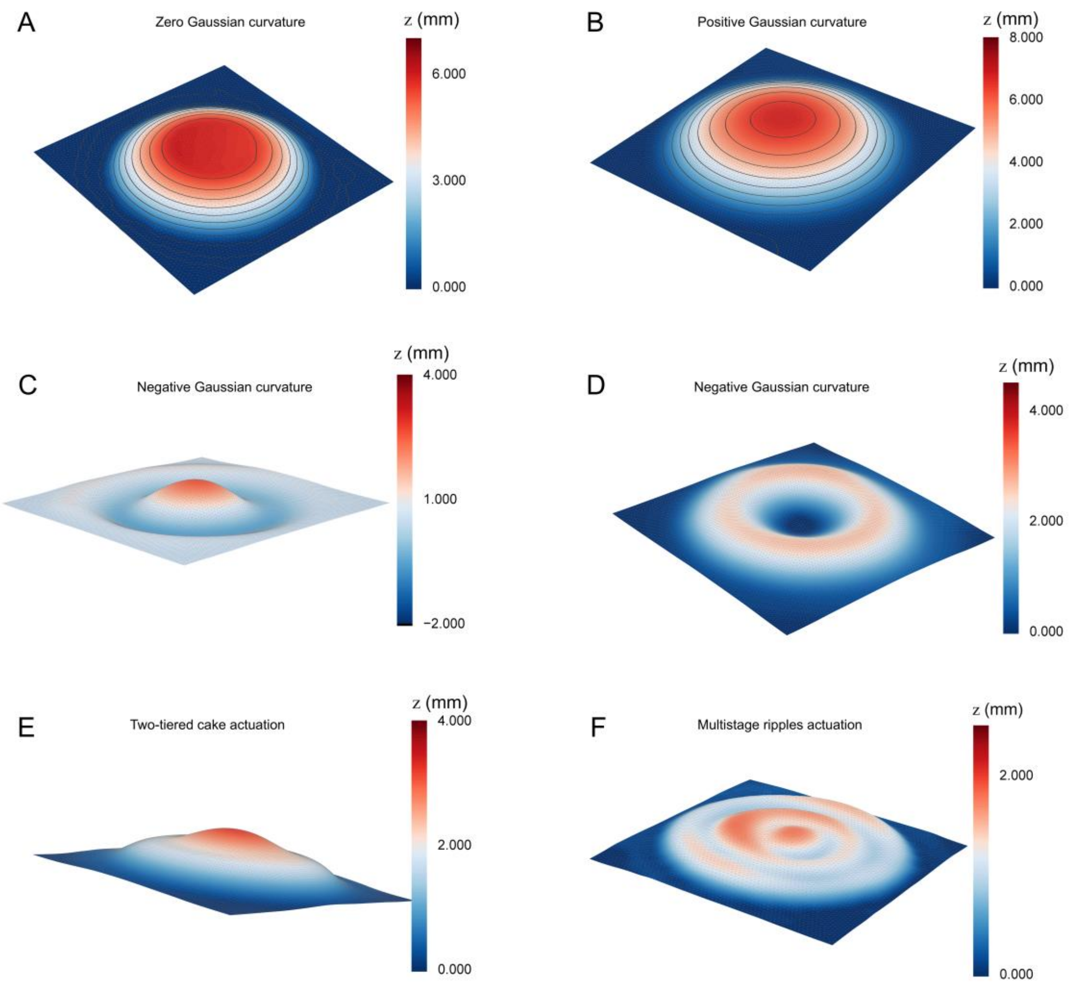

Supplementary Fig. 27 The three-dimensional topography of circular DLCE actuators. **(A)**, Zero Gaussian curvature. **(B)**, Positive Gaussian curvature. **(C)**, Negative Gaussian curvature. **(D)**, Alternative negative Gaussian curvature. **(E)**, Two-tiered cake actuation. **(F)**, Multistage ripples actuation.

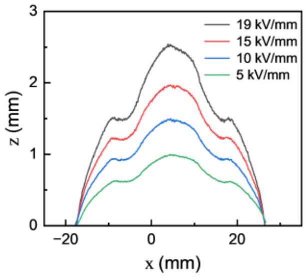

Supplementary Fig. 28 Profiles measurement of the two-tiered cake actuator under different electric fields.

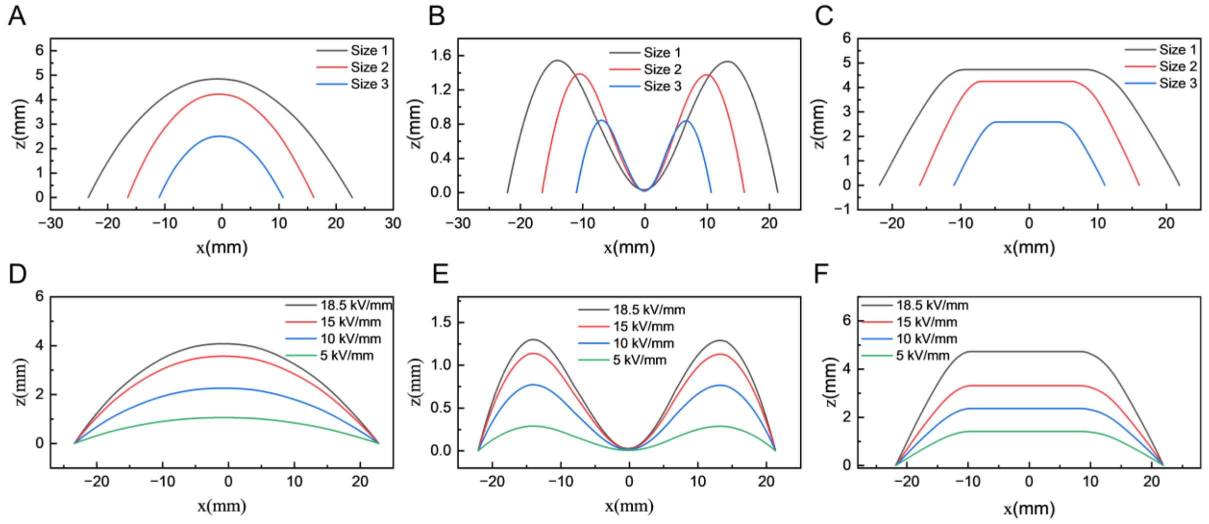

Supplementary Fig. 29 Actuation performance of circular DLCE actuators. (A), Contour profiles of positive Gaussian curvature at varying radii. **(B)**, Contour profiles of negative Gaussian curvature at varying radii. **(C)**, Contour profiles of zero Gaussian curvature at varying radii. **(D)**. Voltage-dependent profiles of positive Gaussian curvature. **(E)**, Voltage-dependent profiles of negative Gaussian curvature. **(F)**, Voltage-dependent profiles of zero Gaussian curvature. (Data points were originally collected at 0.04 mm intervals. To mitigate material surface-smoothness-induced noise, the dataset was smoothed by subsampling at 2 mm intervals.)

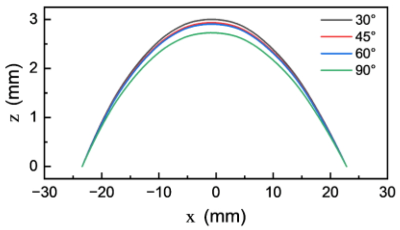

Supplementary Fig. 30 Centroid angular-dependent actuation morphology in convex curvature actuators.

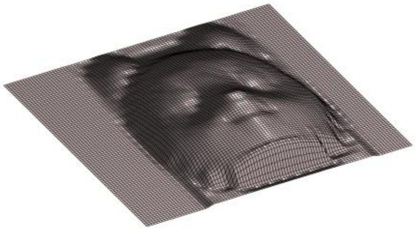

Supplementary Fig. 31. Discretized digital coordinate information set (*x*, *y*, *z*) of the target shape.

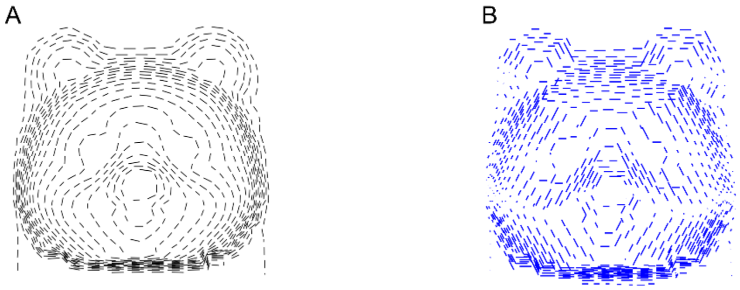

Supplementary Fig. 32. **Schematic diagram for discretizing continuous contour lines into alignment directions of liquid crystal mesogens. (A)** Original segments discretized from continuous contour lines. **(B)** Oriented segments indicating the field of nematic directors.

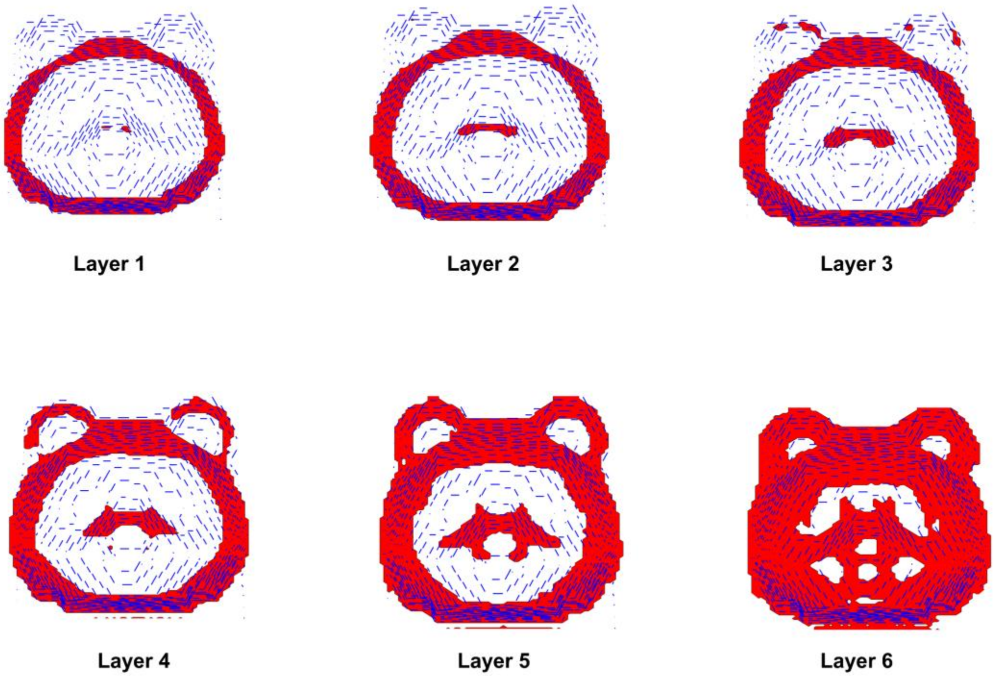

Supplementary Fig. 33. The pattern of monodomain DLCEs in each layer.

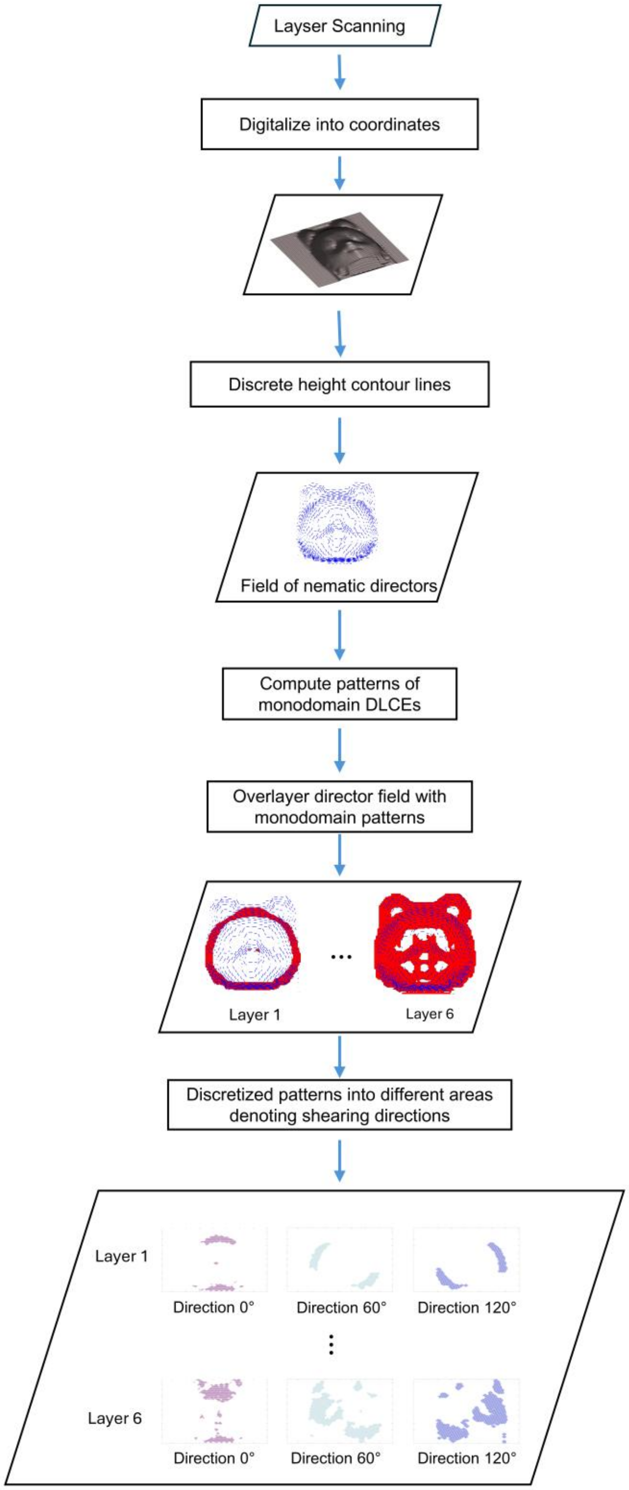

Supplementary Fig. 34 Program flow chart of inverse design method, taking the panda face shape as an example.

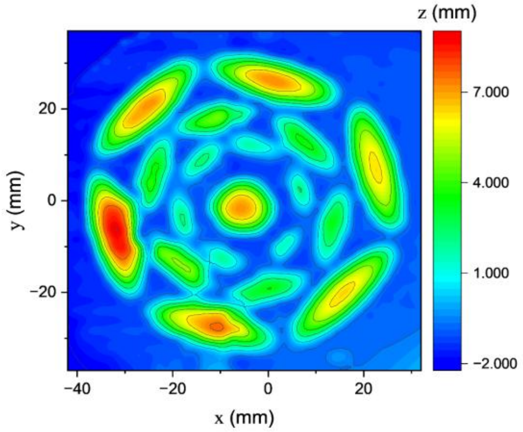

Supplementary Fig. 35 Nineteen closed contour loops of a Graptoveria Amethorum plant.

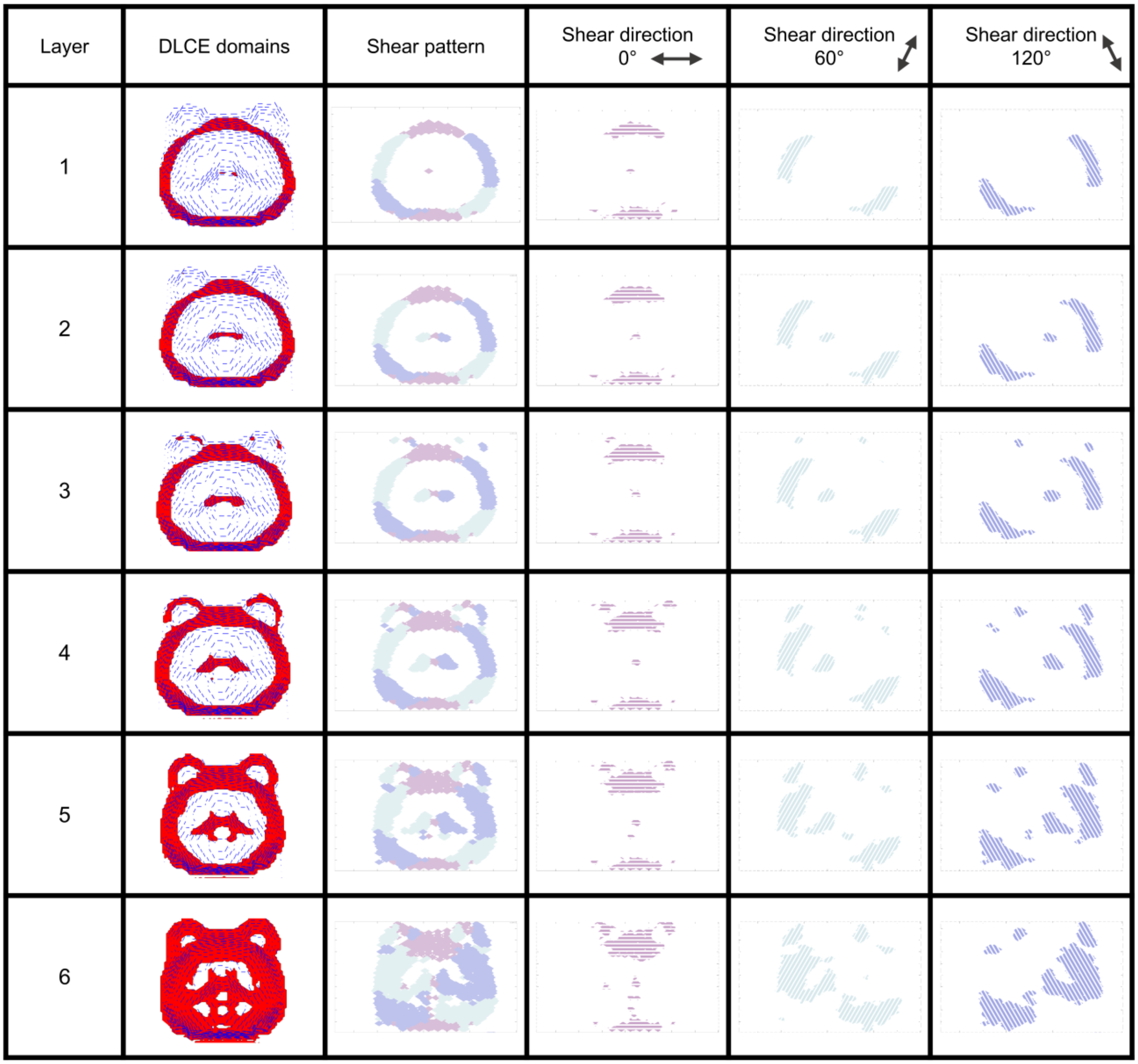

Supplementary Fig. 36 Shear patterns of the panda shape. Shear patterns of the panda shape for each layer of monodomain DLCEs. The second column shows monodomain DLCE overlayed by the nematic director field. The third column shows the merged shearing patterns of each layer with three colors denoting three shearing directions. The fourth to sixth columns show the shearing patterns on each layer with specific directions shown in the header of the table.

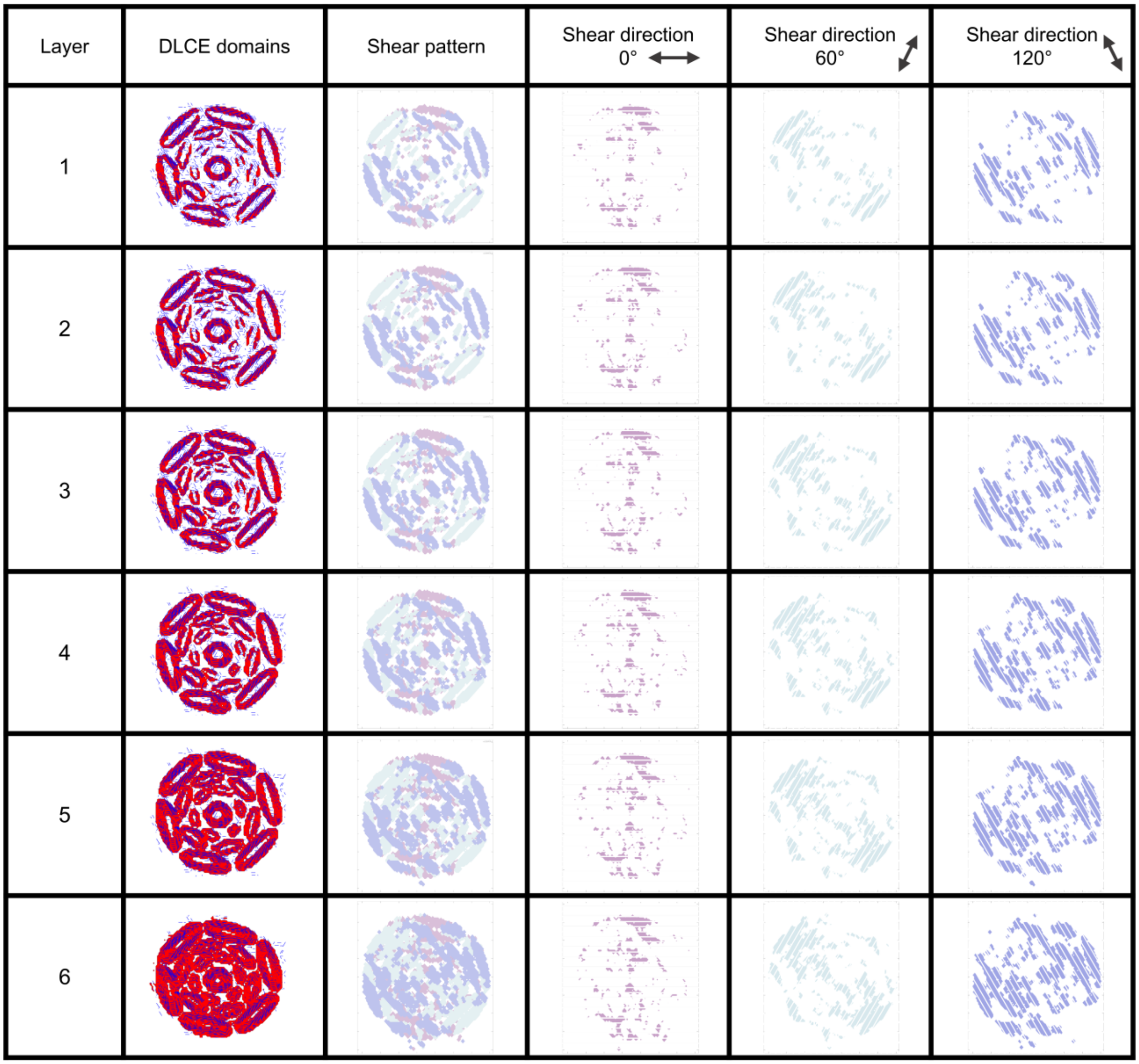

Supplementary Fig. 37 Shear patterns of the Graptoveria Amethorum plant shape. Shear patterns of the Graptoveria Amethorum plant shape for each layer of monodomain DLCEs. The second column shows the merged shearing patterns of each layer with three colors denoting three shearing directions. The third column shows the merged shearing patterns of each layer with three colors denoting three shearing directions. The fourth to sixth columns show the shearing patterns on each layer with specific directions written in the header of the table.

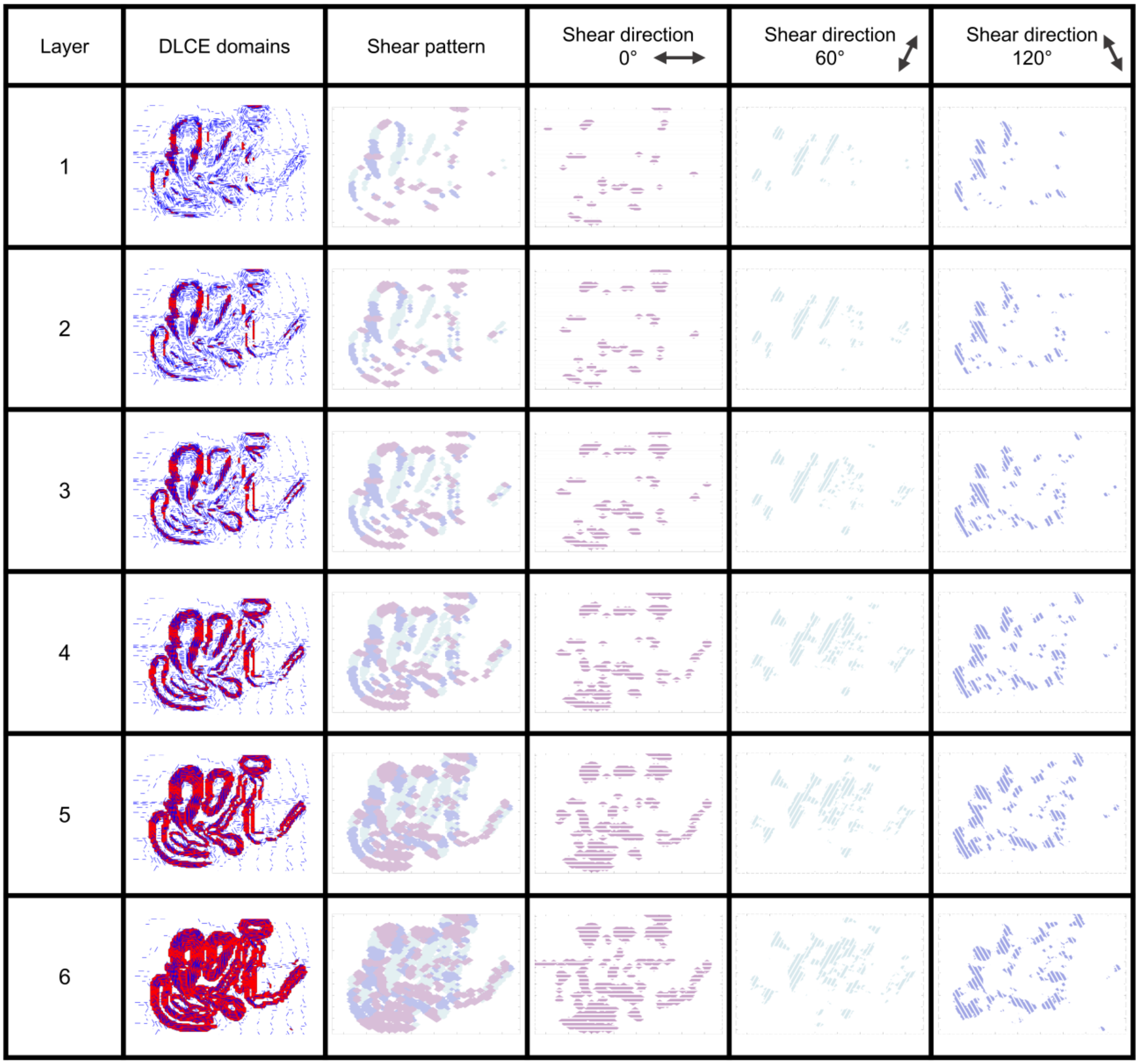

Supplementary Fig. 38 Shear patterns of the Yellow River landform shape. Shear patterns of the Yellow River landform shape for each layer of monodomain DLCEs. The second column shows monodomain DLCE overlayed by the nematic director field. The third column shows the merged shearing patterns of each layer with three colors denoting three shearing directions. The fourth to sixth columns show the shearing patterns on each layer with specific directions in the header of the table. The last column shows the merged shearing patterns of each layer with three colors denoting three shearing directions.

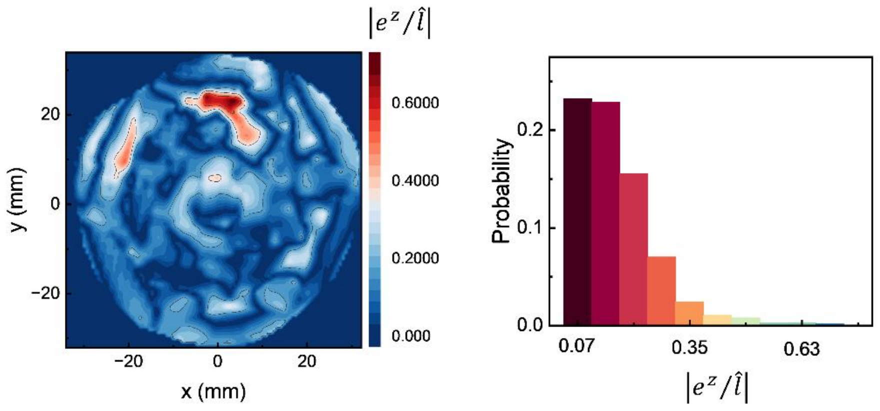

Supplementary Fig. 39 Quantitative characterization of spatial programming errors. **(A)** Contour plots of the absolute error for the out-of-plane displacement, $e^z = z_{actuation} - z_{target}$, normalized by the maximum height of the target shape as the length scale $\hat{l}$. **(B)** The probability distribution of the error, neglecting the zero displacement points ($z_{target} < 0.01$ mm).

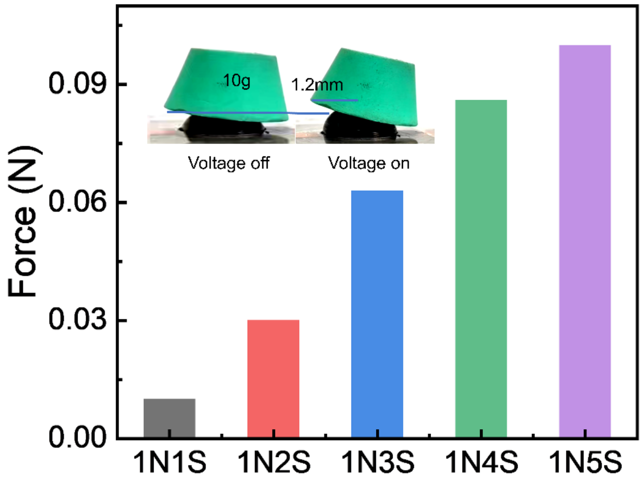

Supplementary Fig. 40 Output force of actuators with different stackingconfigurations (N: Nonsheared, S: Sheared).

Supplementary Movie 1 The electroactive deformation of a bending actuator. The driving morphology of the bending actuator under an electric field intensity of 22.5 kV/mm, with the experimental results on the left and the simulation results on the right.

Supplementary Movie 2 The electroactive deformation of developable surfaced actuators. The driving morphology of complex deformation in a developable surface under an electric field intensity of 20 kV/mm, with the experimental results on the left and the simulation results on the right. The 'S' shape actuator. The twisting actuator. The pyramid actuator. The flower actuator. A windmill actuator.

Supplementary Movie 3 The electroactive deformation of non-developable surfaced actuators. The driving morphology of complex deformation in a non-developable surface under an electric field intensity of 18 kV/mm, with the experimental results on the left and the simulation results on the right. Zero Gaussian curvature. Positive Gaussian curvature. Negative Gaussian curvature. Alternative negative Gaussian curvature. Two-tiered cake actuation. Multistage ripples actuation.

Supplementary Movie 4 The electroactive deformation of complex shape-morphing actuators. The driving morphology of the complex surface deformation under an electric field intensity of 22.5 kV/mm. A panda face shape. The left side shows the front view, and the right side shows the side view. The Graptoveria Amethorum (GA) shape in nature. The shape of the landforms of the Yellow River bend.

REFERENCES AND NOTES

1. A. Sydney Gladman, E. Matsumoto, R. Nuzzo, L. Mahadevan, J. A. Lewis, Biomimetic 4D printing. *Nat. Mater.* **15**, 413–418 (2016).

2. A. J. Ijspeert, Biorobotics: Using robots to emulate and investigate agile locomotion. *Science* **346**, 196–203 (2014).

3. E. E. Kuchen, S. Fox, P. Barbier De Reuille, R. Kennaway, S. Bensmihen, J. Avondo, G. M. Calder, P. Southam, S. Robinson, A. Bangham, E. Coen, Generation of leaf shape through early patterns of growth and tissue polarity. *Science* **335**, 1092–1096 (2012).

4. S. Armon, E. Efrati, R. Kupferman, E. Sharon, Geometry and mechanics in the opening of chiral seed pods. *Science* **333**, 1726–1730 (2011).

5. S.-J. Park, M. Gazzola, K. S. Park, S. Park, V. D. Santo, E. L. Blevins, J. U. Lind, P. H. Campbell, S. Dauth, A. K. Capulli, F. S. Pasqualini, S. Ahn, A. Cho, H. Yuan, B. M. Maoz, R. Vijaykumar, J.-W. Choi, K. Deisseroth, G. V. Lauder, L. Mahadevan, K. K. Parker, Phototactic guidance of a tissue-engineered soft-robotic ray. *Science* **353**, 158–162 (2016).

6. Z. Xue, T. Jin, S. Xu, K. Bai, Q. He, F. Zhang, X. Cheng, Z. Ji, W. Pang, Z. Shen, H. Song, Y. Shuai, Y. Zhang, Assembly of complex 3D structures and electronics on curved surfaces. *Sci. Adv.* **8**, eabm6922 (2022).

7. D. Li, Y. Sun, X. Li, X. Li, Z. Zhu, B. Sun, S. Nong, J. Wu, T. Pan, W. Li, S. Zhang, M. Li, 3D printing of near-ambient responsive liquid crystal elastomers with enhanced nematic order and pluralized transformation. *ACS Nano* **19**, 7075–7087 (2025).

8. Y. Zhu, N. Liu, Z. Chen, H. He, Z. Wang, Z. Gu, Y. Chen, J. Mao, Y. Luo, Y. He, 3D-printed high-frequency dielectric elastomer actuator toward insect-scale ultrafast soft robot. *ACS Mater. Lett.* **5**, 704–714 (2023).

9. A. Chortos, J. Mao, J. Mueller, E. Hajiesmaili, J. A. Lewis, D. R. Clarke, Printing reconfigurable bundles of dielectric elastomer fibers. *Adv. Funct. Mater.* **31**, 2010643 (2021).

10. Y. Kim, H. Yuk, R. Zhao, S. A. Chester, X. Zhao, Printing ferromagnetic domains for untethered fast-transforming soft materials. *Nature* **558**, 274–279 (2018).

11. X. Cheng, Z. Fan, S. Yao, T. Jin, Z. Lv, Y. Lan, R. Bo, Y. Chen, F. Zhang, Z. Shen, H. Wan, Y. Huang, Y. Zhang, Programming 3D curved mesosurfaces using microlattice designs. *Science* **379**, 1225–1232 (2023).

12. M. Han, X. Guo, X. Chen, C. Liang, H. Zhao, Q. Zhang, W. Bai, F. Zhang, H. Wei, C. Wu, Q. Cui, S. Yao, B. Sun, Y. Yang, Q. Yang, Y. Ma, Z. Xue, J. W. Kwak, T. Jin, Q. Tu, E. Song, Z. Tian, Y. Mei, D. Fang, H. Zhang, Y. Huang, Y. Zhang, J. A. Rogers, Submillimeter-scale multimaterial terrestrial robots. *Sci. Robot.* **7**, eabn0602 (2022).

13. B. H. Kim, K. Li, J. Kim, Y. Park, H. Jang, X. Wang, Z. Xie, S. M. Won, H. Yoon, G. Lee, W. J. Jang, K. H. Lee, T. S. Chung, Y. H. Jung, S. Y. Heo, Y. Lee, J. Kim, T. Cai, Y. Kim, P. Prasopsukh, Y. Yu, X. Yu, R. Avila, H. Luan, H. Song, F. Zhu, Y. Zhao, L. Chen, S. H. Han, J. Kim, S. J. Oh, H. Lee, C. H. Lee, Y. Huang, L. P. Chamorro, Y. Zhang, J. A. Rogers, Three-dimensional electronic microfliers inspired by wind-dispersed seeds. *Nature* **597**, 503–510 (2021).

14. C. Zhang, C. Pan, K. F. Chan, J. Gao, Z. Yang, K. K. C. Leung, D. Jin, Y. Wang, N. Xia, Z. Ning, X. Wang, S. Jiang, Z. Zhang, Q. Wang, B. Hao, P. W. Y. Chiu, L. Zhang, Wirelessly powered deformable electronic stent for noninvasive electrical stimulation of lower esophageal sphincter. *Sci. Adv.* **9**, eade8622 (2023).

15. A. Nojoomi, J. Jeon, K. Yum, 2D material programming for 3D shaping. *Nat. Commun.* **12**, 603 (2021).

16. E. Siéfert, E. Reyssat, J. Bico, B. Roman, Bio-inspired pneumatic shape-morphing elastomers. *Nat. Mater.* **18**, 24–28 (2019).

17. N. Xia, D. Jin, C. Pan, J. Zhang, Z. Yang, L. Su, J. Zhao, L. Wang, L. Zhang, Dynamic morphological transformations in soft architected materials via buckling instability encoded heterogeneous magnetization. *Nat. Commun.* **13**, 7514 (2022).

18. D. Jin, Q. Chen, T. Huang, J. Huang, L. Zhang, H. Duan, Four-dimensional direct laser writing of reconfigurable compound micromachines. *Mater. Today* **32**, 19–25 (2020).

19. T. Y. Huang, H. W. Huang, D. D. Jin, Q. Y. Chen, J. Y. Huang, L. Zhang, H. L. Duan, Four-dimensional micro-building blocks. *Sci. Adv.* **6**, eaav8219 (2020).

20. T. Xu, J. Zhang, M. Salehizadeh, O. Onaizah, E. Diller, Millimeter-scale flexible robots with programmable three-dimensional magnetization and motions. *Sci. Robot.* **4**, eaav4494 (2019).

21. Y. Dong, L. Wang, N. Xia, Z. Yang, C. Zhang, C. Pan, D. Jin, J. Zhang, C. Majidi, L. Zhang, Untethered small-scale magnetic soft robot with programmable magnetization and integrated multifunctional modules. *Sci. Adv.* **8**, eabn8932 (2022).

22. J. Zhang, Z. Ren, W. Hu, R. H. Soon, I. C. Yasa, Z. Liu, M. Sitti, Voxelated three-dimensional miniature magnetic soft machines via multimaterial heterogeneous assembly. *Sci. Robot.* **6**, eabf0112 (2021).

23. R. Bo, S. Xu, Y. Yang, Y. Zhang, Mechanically-guided 3D assembly for architected flexible electronics. *Chem. Rev.* **123**, 11137–11189 (2023).

24. R. Sawhney, K. Crane, Boundary first flattening. *ACM Trans. Graph.* **37**, 1–14 (2018).

25. X. Sun, L. Yue, L. Yu, C. T. Forte, C. D. Armstrong, K. Zhou, F. Demoly, R. R. Zhao, H. J. Qi, Machine learning-enabled forward prediction and inverse design of 4D-printed active plates. *Nat. Commun.* **15**, 5509 (2024).

26. T. Gao, J. Bico, B. Roman, Pneumatic cells toward absolute Gaussian morphing. *Science* **381**, 862–867 (2023).

27. J. H. Pikul, S. Li, H. Bai, R. T. Hanlon, I. Cohen, R. F. Shepherd, Stretchable surfaces with programmable 3D texture morphing for synthetic camouflaging skins. *Science* **358**, 210–214 (2017).

28. Y. Bai, H. Wang, Y. Xue, Y. Pan, J. Kim, X. Ni, T. Liu, Y. Yang, M. Han, Y. Huang, J. A. Rogers, X. Ni, A dynamically reprogrammable surface with self-evolving shape morphing. *Nature* **609**, 701–708 (2022).

29. A. Nojoomi, H. Arslan, K. Lee, K. Yum, Bioinspired 3D structures with programmable morphologies and motions. *Nat. Commun.* **9**, 3705 (2018).

30. Y. Ling, W. Pang, J. Liu, M. Page, Y. Xu, G. Zhao, D. Stalla, J. Xie, Y. Zhang, Z. Yan, Bioinspired elastomer composites with programmed mechanical and electrical anisotropies. *Nat. Commun.* **13**, 524 (2022).

31. N. Xia, D. Jin, Z. Yang, C. Pan, L. Su, M. Zhang, X. Wang, Z. Xu, Z. Guo, L. Pan, X. Sun, X. Ma, L. Wang, L. Zhang, Inverse programming of ferromagnetic domains for 3D curved surfaces of soft materials. *Nat. Synth.* **4**, 642–654 (2025).

32. M. Duduta, R. J. Wood, D. R. Clarke, Multilayer dielectric elastomers for fast, programmable actuation without prestretch. *Adv. Mater.* **28**, 8058–8063 (2016).

33. H. Yang, X. Yin, C. Zhang, B. Chen, P. Sun, Y. Xu, Weaving liquid crystal elastomer fiber actuators for multifunctional soft robotics. *Sci. Adv.* **11**, eads3058 (2025).

34. S. Oh, C. Y. Kim, S. Chung, J. Jeong, Cephalopod-inspired magnetic shape-morphing system for complex 3D transformations with broad reconfigurability in 3D displays and soft robotics. *Adv. Mater.* **37**, e2417913 (2025).

35. J. Pu, Y. Meng, Z. Xie, Z. Peng, J. Wu, Y. Shi, R. Plamthottam, W. Yang, Q. Pei, A unimorph nanocomposite dielectric elastomer for large out-of-plane actuation. *Sci. Adv.* **8**, eabm6200 (2022).

36. S. Wei, T. K. Ghosh, Bioinspired bistable dielectric elastomer actuators: Programmable shapes and application as binary valves. *Soft Robot.* **9**, 900–906 (2022).

37. X. Zhao, Z. Suo, Method to analyze programmable deformation of dielectric elastomer layers. *Appl. Phys. Lett.* **93**, 251902 (2008).

38. E. Hajiesmaili, N. M. Larson, J. A. Lewis, D. R. Clarke, Programmed shape-morphing into complex target shapes using architected dielectric elastomer actuators. *Sci. Adv.* **8**, eabn9198 (2022).

39. B. Aksoy, H. Shea, Reconfigurable and latchable shape-morphing dielectric elastomers based on local stiffness modulation. *Adv. Funct. Mater.* **30**, 2001597 (2020).

40. E. Hajiesmaili, D. R. Clarke, Reconfigurable shape-morphing dielectric elastomers using spatially varying electric fields. *Nat. Commun.* **10**, 183 (2019).

41. J. Shintake, V. Cacucciolo, H. Shea, D. Floreano, Soft biomimetic fish robot made of dielectric elastomer actuators. *Soft Robot.* **5**, 466–474 (2018).

42. E. Hajiesmaili, E. Khare, A. Chortos, J. Lewis, D. R. Clarke, Voltage-controlled morphing of dielectric elastomer circular sheets into conical surfaces. *Extreme Mech. Lett.* **30**, 100504 (2019).

43. S. Li, H. Bai, Z. Liu, X. Zhang, C. Huang, L. W. Wiesner, M. Silberstein, R. F. Shepherd, Digital light processing of liquid crystal elastomers for self-sensing artificial muscles. *Sci. Adv.* **7**, eabg3677 (2021).

44. Z. S. Davidson, H. Shahsavan, A. Aghakhani, Y. Guo, L. Hines, Y. Xia, S. Yang, M. Sitti, Monolithic shape-programmable dielectric liquid crystal elastomer actuators. *Sci. Adv.* **5**, eaay0855 (2019).

45. K. M. Herbert, H. E. Fowler, J. M. Mccracken, K. R. Schlafmann, J. A. Koch, T. J. White, Synthesis and alignment of liquid crystalline elastomers. *Nat. Rev. Mater.* **7**, 23–38 (2022).

46. Q. Ze, S. Wu, J. Nishikawa, J. Dai, Y. Sun, S. Leanza, C. Zemelka, L. S. Novelino, G. H. Paulino, R. R. Zhao, Soft robotic origami crawler. *Sci. Adv.* **8**, eabm7834 (2022).

47. J. Wang, A. Chortos, Performance metrics for shape-morphing devices. *Nat. Rev. Mater.* **9**, 738–751 (2024).

48. R. Xiao, Z. Chen, Y. Shi, L. Zhan, S. Qu, P. Steinmann, A continuum model for novel electromechanical-instability-free dielectric elastomers. *J. Mech. Phys. Solids* **196**, 105994 (2025).

49. E. Allahyari, M. Asgari, Fiber reinforcement characteristics of anisotropic dielectric elastomers: A constitutive modeling development. *Mech. Adv. Mater. Struct.* **29**, 5542–5556 (2022).

50. H. E. Fowler, P. Rothemund, C. Keplinger, T. J. White, Liquid crystal elastomers with enhanced directional actuation to electric fields. *Adv. Mater.* **33**, 2103806 (2021).

51. M. O. Saed, C. P. Ambulo, H. Kim, R. De, V. Raval, K. Searles, D. A. Siddiqui, J. M. O. Cue, M. C. Stefan, M. R. Shankar, T. H. Ware, Molecularly-engineered, 4D-printed liquid crystal elastomer actuators. *Adv. Funct. Mater.* **29**, 1806412 (2019).

52. M. O. Saed, R. H. Volpe, N. A. Traugutt, R. Visvanathan, N. A. Clark, C. M. Yakacki, High strain actuation liquid crystal elastomers via modulation of mesophase structure. *Soft Matter* **13**, 7537–7547 (2017).

53. D. S. Shah, E. J. Yang, M. C. Yuen, E. C. Huang, R. Kramer-Bottiglio, Jamming skins that control system rigidity from the surface. *Adv. Funct. Mater.* **31**, 2006915 (2021).

54. M. Coelho, H. Ishii, P. Maes, Surflex: A programmable surface for the design of tangible interfaces, in *CHI'08 Extended Abstracts on Human Factors in Computing Systems* (2008), 3429–3434.

55. B. Yang, R. Baines, D. Shah, S. Patiballa, E. Thomas, M. Venkadesan, R. Kramer-Bottiglio, Reprogrammable soft actuation and shape-shifting via tensile jamming. *Sci. Adv.* **7**, eabh2073 (2021).

56. E. Steltz, A. Mozeika, N. Rodenberg, E. Brown, H. M. Jaeger, JSEL: Jamming skin enabled locomotion, in *2009 IEEE/RSJ International Conference on Intelligent Robots and Systems* (IEEE, 2009), pp. 5672–5677.

57. C. Li, G. C. Lau, H. Yuan, A. Aggarwal, V. L. Dominguez, S. Liu, H. Sai, L. C. Palmer, N. A. Sather, T. J. Pearson, D. E. Freedman, P. K. Amiri, M. O. de la Cruz, S. I. Stupp, Fast and programmable locomotion of hydrogel-metal hybrids under light and magnetic fields. *Sci. Robot.* **5**, eabb9822 (2020).

58. M. E. Mcconney, A. Martinez, V. P. Tondiglia, K. M. Lee, D. Langley, I. I. Smalyukh, T. J. White, Topography from topology: Photoinduced surface features generated in liquid crystal polymer networks. *Adv. Mater.* **25**, 5880–5885 (2013).

59. K. Liu, F. Hacker, C. Daraio, Robotic surfaces with reversible, spatiotemporal control for shape morphing and object manipulation. *Sci. Robot.* **6**, eabf5116 (2021).

60. J. W. Boley, W. M. van Rees, C. Lissandrello, M. N. Horenstein, R. L. Truby, A. Kotikian, J. A. Lewis, L. Mahadevan, Shape-shifting structured lattices via multimaterial 4D printing. *Proc. Natl. Acad. Sci. U.S.A.* **116**, 20856–20862 (2019).

61. J. Wang, M. Sotzing, M. Lee, A. Chortos, Passively addressed robotic morphing surface (PARMS) based on machine learning. *Sci. Adv.* **9**, DOI: 10.1126/sciadv.adg8019 (2023).

62. X. Ni, H. Luan, J.-T. Kim, S. I. Rogge, Y. Bai, J. W. Kwak, S. Liu, D. S. Yang, S. Li, S. Li, Z. Li, Y. Zhang, C. Wu, X. Ni, Y. Huang, H. Wang, J. A. Rogers, Soft shape-programmable surfaces by fast electromagnetic actuation of liquid metal networks. *Nat. Commun.* **13**, 5576 (2022).

63. A. M. Rauf, J. S. Bernardo, S. Follmer, Electro adhesive auxetics as programmable layer jamming skins for formable crust shape displays. IEEE Access 11 Mar. 2023.
